# Bionic bearing-inspired lubricating microspheres with Immunomodulatory effects for osteoarthritis therapy

**DOI:** 10.1186/s12951-025-03544-2

**Published:** 2025-06-20

**Authors:** Xu He, Xingzhi Liu, Xinyi Cheng, Can Zhu, Yida Chen, Yi Wang, Yun Zhou, Hao Shen, Huilin Yang, Yong Xu, Qin Shi, Junjie Niu

**Affiliations:** 1 Department of Orthopedics, The First Affiliated Hospital of Soochow University, Orthopedic Institute of Soochow University, Medical College of Soochow University, 899 Pinghai Road, Suzhou, 215031 Jiangsu P. R. China; 2 Rui Jin Hospital, Lu Wan Branch, Shanghai Jiao Tong University School of Medicine, NO.149 South Chongqing Road, Shanghai, 200000 P. R. China

**Keywords:** Osteoarthritis, Microspheres, Cartilage adhesion, Inflammatory, Sericin methacryloyl, Chondroitin sulfate

## Abstract

Inflammatory infiltration and inappropriate friction remain the two main challenges in treating osteoarthritis (OA). An effective treatment platform is anticipated to lubricate the cartilage and ameliorate intra-articular inflammation. Herein, injectable hydrogel microspheres with chondroitin sulfate (Chs)/sericin methacryloyl (SerMA) designed as the core and grafted with folic acid (FA)- and 78c-modified liposomes (78c@Lipo-FA) by DSPE-PEG2k-GPLGLAGQC (DPG) peptides, namely LFDCS, was formed by thin-film hydration and microfluidic methods. Physically, LFDCS exhibited cartilage lubrication and adhesion capability as the bionic bearings. Meanwhile, with the degradation of LFDCS, Chs were set out to promote chondrocyte function. Physiologically, 78c@Lipo-FA were released from LFDCS enzymatically responsible to matrix metalloprotein-9 (MMP9) due to the split of DPG motif, which specifically bound to classical-activated macrophages (M1) by ligating to FA receptor through FA and inhibit M1 function by 78c, consequently rescued cartilage from degradation by inflammation. Mechanically, an RNA sequencing analysis revealed that the LFDCS could ameliorate chondrocyte dysfunction by down-regulating PI3K/AKT signaling and inflammation response. LFDCS greatly reduced friction on articular surfaces, suppressed M1-induced inflammation and attenuated chondrocyte dysfunction in OA rats. In conclusion, the bionic bearing-inspired lubricating microspheres characterized by M1-phenotype macrophage-targeted addresses the unfavorable factors of both the physical and physiological microenvironments in macrophage-mediated diseases.

**Graphic abstract:**

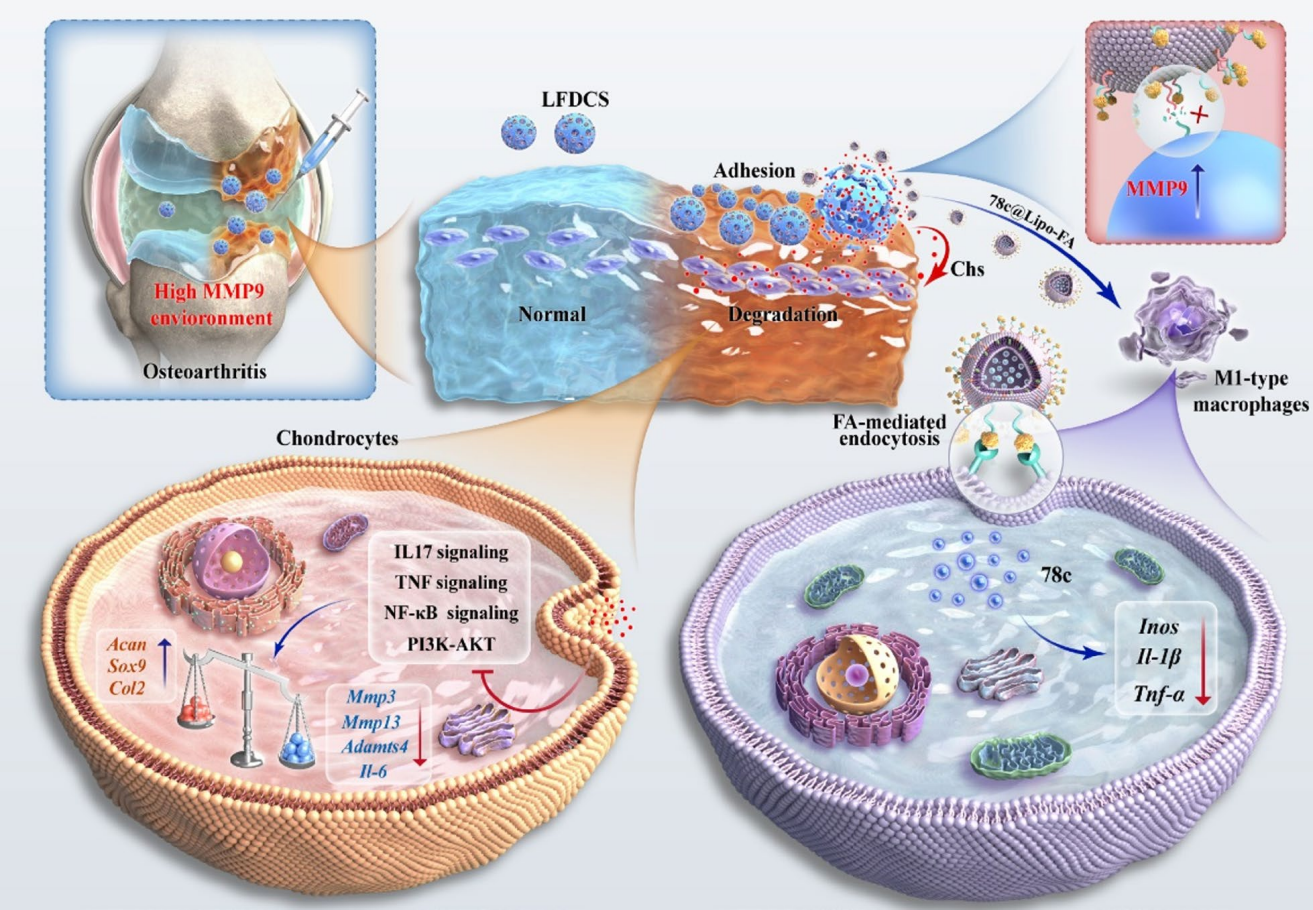

**Supplementary Information:**

The online version contains supplementary material available at 10.1186/s12951-025-03544-2.

## Introduction

Hydrogel microspheres, characterized by outstanding injectability and biocompatibility, are versatile drug delivery systems extensively applied to regenerate bone, cartilage, dental, nerve, cardiac, and skin tissues [1]. Hydrogel microspheres function as ball bearings, minimize friction during interfacial contact and effectively deliver drugs to the injury sites [2]. Although, commonly used microsphere materials (gelatin, chitosan, and hyaluronic acid [HA]) cover the entire repair area for protection, the microspheres are present in micrometer-sized motile forms and may accumulate in non-action areas during movement/friction. This not only reduces their effectiveness, but also reduces the effectiveness of the medication [3–5]. Currently, the primary approach to improve microsphere adhesion to tissues and cells involves chemical modifications such as thiolation-modified HA microspheres that bind to the intestinal mucosa and enhance microsphere adhesion [6]. Dopamine-modified chitosan hydrogels are employed to improve cell adhesion for treating spinal cord injuries [7], while dopamine-modified HA microspheres and catecholamine-modified chondroitin sulfate (Chs) hydrogels can adhere firmly to the articular surface and improve the efficiency of drug enrichment on the cartilage surface [3, 4]. However, excessive chemical modification may decrease biocompatibility and increase the immunogenicity of the material [8]. The development of new natural biomaterials with biocompatibility and tissue adhesion is crucial for tissue regeneration engineering.

Sericin is a water-soluble glycoprotein derived from silkworm cocoons that comprises 18 amino acids [9]. Methacrylic anhydride (MA)-modified sericin (SerMA) solutions with remarkable injectability and plasticity have potential use in regenerative repair [10, 11]. A porous double-crosslinked hydrogel containing sericin has been found to enhance the synthesis of natural cartilage-like tissue by facilitating chondrocyte adhesion and proliferation while mitigating inflammatory cell infiltration [12]. Lin et al. promoted drug penetration into the cartilage matrix by dopamine-modified structures on the surface of microspheres, which could effectively attach the microspheres to the cartilage surface [4]. However, after the introduction of dopamine into hydrogels, the catechol structure of dopamine is easily oxidized by the microenvironment, which significantly reduces the adhesion stability and limits the practical application of hydrogels [13]. Sericin contain large amounts of serine, the hydroxyl group of its side chain (-OH) can form extensive hydrogen bonding, thus providing adhesion, which is conducive to the physical adhesion of the interface [14]. SerMA is widely used in the treatment of diseases with variable local microenvironments and requiring precise adhesion for drug delivery due to its physical properties and stable adhesion properties.

OA is a progressive disease caused by inflammation and cartilage wear and tear [15, 16]. Excessive inflammation and the difficulty of conventional delivery systems to improve local drug enrichment are key issues that make it difficult to progress in the treatment of OA [17, 18]. Lin et al. pointed out that intra-articular drug delivery is susceptible to rapid clearance by synovial capillaries and lymphatics, limiting intra-articular drug administration [19]. Therefore, there is a need to shorten the cartilage repair drug delivery distance to achieve high drug concentration enrichment at the injury. Additionally, macrophages are among the plentiful non-specific immune cells in the synovium, and they are integral to the initiation and exacerbation of OA through their involvement in the initial inflammatory response [20–23]. M1 macrophages exacerbate OA progression in mice by regulating R-spondin-2 [24]. Xiao et al. reprogrammed macrophages by developing a CD86-targeted microsphere for the treatment of OA [25]. Macrophage polarization is a continuous process that presents a mixed phenotype (i.e., expressing both CD86 and CD206 ) in complex microenvironments [26]. However, current targeting strategies fail to selectively target M1 macrophages without impacting other bone marrow lineages (dendritic cells and neutrophils) [22]. CD38, a type II transmembrane protein, is pivotal in regulating macrophage function [27]. Recent findings have indicated that CD38 is highly expressed exclusively in M1 macrophages, whereas its expression is low in M0 and M2 macrophages [28]. The knockdown of CD38 or localized administration of CD38 inhibitors (apigenin or 78c) delays cartilage degeneration and subchondral bone sclerosis in mouse joints [29, 30]. Therefore, focusing on the inflammatory management of macrophages in the early stages of OA, aiming at restoring homeostasis of the local immune microenvironment and promoting articular cartilage lubrication, is a promising therapeutic strategy for OA.

**Scheme 1:**
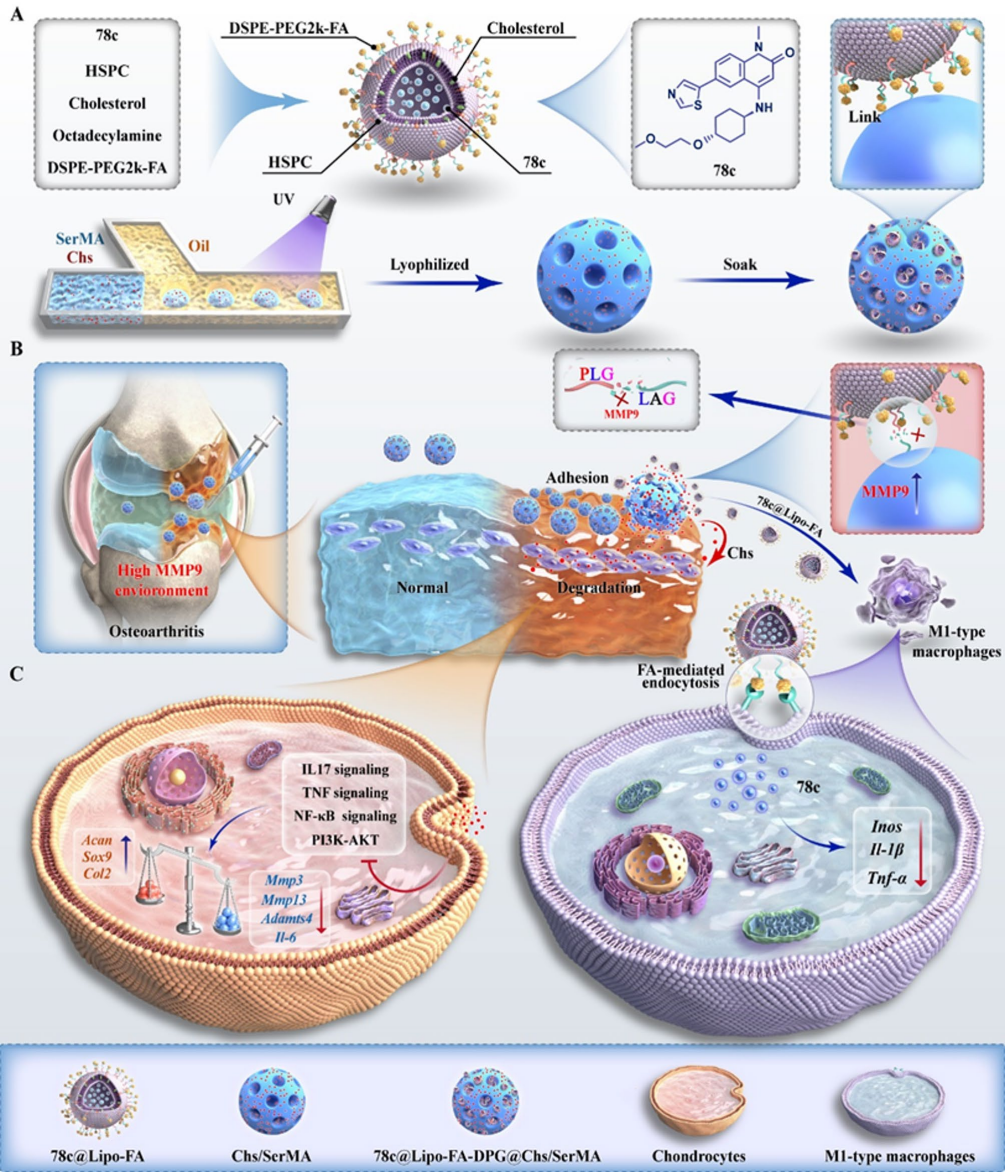
Bionic bearing-inspired lubricating microspheres characterized by immunomodulatory for osteoarthritis treatment. (**A**) Synthesis of M1-targeted liposomes and highly adhesive lubricating hydrogel microspheres. (**B**) Application of composite hydrogel microspheres in OA. (**C**) Mechanism of composite hydrogel microspheres to ameliorate chondrocyte dysfunction through downregulation of inflammatory and PI3K/AKT signaling pathways and inhibit M1-mediated inflammation. Ultraviolet: UV

The lack of joint lubrication, the pathological features of inflammatory imbalance and the difficulty of local drug enrichment are key issues in the treatment of OA. Herein, we used thin-film aqueous and microfluidic techniques with the aim of developing an effective physically- and physiologically-coupled matrix metallopeptidase (MMP)-responsive hydrogel microspheres drug delivery system that mitigates OA. We prepared folic acid (FA)- and 78c- modified liposomes (78c@Lipo-FA). Then, we anchored 78c@Lipo-FA to the injectable chondroitin sulfate (Chs)/sericin methacryloyl (SerMA) hydrogel microspheres with DSPE-PEG2k-GPLGLAGQC (DPG) peptides (78c@Lipo-FA-DPG@Chs/SerMA hydrogel microspheres [LFDCS]). This innovative approach enabled the LFDCS coupled with physiological and physical characters to rescue OA in rats (Scheme 1A). LFDCS physically lubricate articular cartilage and adhere to articular surfaces, and further increase local drug concentration to promote chondrocyte function. Physiologically, upon an MMP9 environment, for example, in OA, MMP9 cleaved DPG leading to LFDCS to set out 78c@Lipo-FA, which effectively targeted M1-phenotype macrophages by ligating to FA receptors through FA and inhibited M1-mediated inflammation by 78c, consequently to alleviated OA processing (Scheme 1B). Therefore, we successfully developed injectable bionic bearing-inspired lubricating microspheres by coupling physiological and physical characters, that exert the physical action of adherence to articular cartilage and bearing lubricated joints, as well as the physiological action of intelligent targeting of macrophage inflammation and amelioration of chondrocyte dysfunction. This innovative system effectively addresses the challenges of elevated intra-articular inflammation and diminished joint lubrication.

## Experiment section

### Materials

Hydrogenated soybean phospholipid (HSPC), cholesterol, and octadecylamine were purchased from Aladdin (Shanghai, China). DSPE-PEG2k-FA and DAPI were obtained from Yuanye (Shanghai, China). The CD38 inhibitor (78c) was purchased from Selleck (Wuhan, China). The Cell Plasma Membrane Staining Kit with DiO, immunofluorescence permeabilization and containment solutions, 1% penicillin/streptomycin (P/S), calcein/PI cell viability assay kit, 4% paraformaldehyde, and Toluidine blue staining kit were sourced from Beyotime (Shanghai, China). Cy5.5 were obtained from Duofluor (Wuhan, China). SerMA and Chs were sourced from Suzhou Engineering for Life Sciences (Shanghai, China). Sulfo-SMCC and 0.2% Collagenase II were obtained from Sigma (Thermo, USA). DSPE-PEG2k-GPLGLAGQC (DPG) was synthesized by Xi’an Ruixi (Xi’an, China). Lipopolysaccharides (LPS), recombinant rat IL-1β, and recombinant mouse M-CSF were purchased from Peprotech (Cranbury, USA). CD11b-eFluor 450, CD86-PE-Cy7, and CD206-FITC were obtained from Biolegend (San Diego, USA). TRIzol and cell counting kit-8 (CCK-8) were obtained from Dojindo Laboratories (Kumamoto, Japan). The Script RT Kit was sourced from Abcam (Cambridge, UK). SYBR Green was obtained from Bio-Rad (Hercules, USA). Phalloidin was purchased from Invitrogen (Thermo, USA). MEM Alpha medium, DMEM medium, 0.25% trypsin, and fetal bovine serum (FBS) were obtained from Gibco (Thermo, USA). The immunohistochemistry (IHC) kit was sourced from Jiancheng (Nanjing, China). Recombinant anti-agrecan (ACAN) and anti- MMP13 antibodies were purchased from Abcam. MMP9 was purchased from Acros Biosystems (Shanghai, China). Rat IL-6 and TNF-α enzyme-linked-immunosorbent-assay (ELISA) kits were purchased from Elabscience (Wuhan, China). Hematoxylin and eosin (H&E), Safranin O and Fast Green Stain (Safranin O) kits were purchased from SolarBio (Beijing, China).

### Synthesis and characterization of liposomes

Film hydration and extrusion were used to prepare liposomes (Lipo), Lipo-FA, and 78c@Lipo-FA [31]. Briefly, 20 mg HSPC, 5 mg cholesterol, 2 mg octadecylamine, 2.76 mg DSPE-PEG2k-FA, and 0.2 mg 78c were dissolved in 5 mL chloroform to generate a thin-film using evaporator at 65℃. The thin-film was then hydrated with 6 mL double distilled water by stirring for 2 h (h). After ultrasonication of the liposomes for 20 min (min), the liposomes were filtered through 0.4-µm and 0.2-µm filters.

After negative staining of the liposomes, the observation of liposome morphology was conducted by transmission electron microscope (TEM) (Hitachi, Japan). A zetasizer advance nanoparticle analyzer (Malvern, England) was used to detect liposome particle size and zeta potential.

For the preparation of DiO-stained liposomes, 500 µL liposome suspension was subjected to centrifugation at 10,000 g for 30 min to obtain a liposome precipitate. Liposomes were stained with DiO for 10 min according to the instructions.

### Targeting and uptake of bone marrow-derived macrophages (BMMs)

BMMs were obtained from 6-week-old C57BL/6 mice as described previously [32]. BMMs (1 × 10^5^ cells/well) were seeded in a 12-well plate containing MEM Alpha medium. After 24 h of polarization induction (LPS, 100 ng/mL), the MEM Alpha medium was replaced with 10 µL DiO-stained Lipo, Lipo-FA, and 78c@Lipo-FA, repspectively. After BMMs were incubated for 4 h, the cells were digested and the fluorescent intensity of DiO was quantified using a flow cytometer (Thermo, USA). For cell staining, the treated cells were stained with DAPI. Images were taken by an inverted fluorescence microscope (Zeiss, Germany), and quantification was used ImageJ software (Rawak Software Inc., Germany).

### Synthesis and characterization of SerMA hydrogel microspheres

To select the SerMA hydrogel concentration, different concentrations of SerMA (5%, 15%, and 25% w/v) were dissolved in 0.25% 2,4,6-trimethylbenzamide (LAP). A rheometer (TA Instruments, USA) was utilized to detect the changes in elastic modulus (G’) and viscous modulus (G’’) of SerMA hydrogel during the curing process.

SerMA (25% w/v) were dissolved in LAP as the aqueous phase and isopropyl myristate as the oil phase. The aqueous and oil phases flowed at different rates in the microfluidic device and were cured into hydrogel microspheres under UV light [33]. Ethanol and double distilled water were used to clean cured microspheres. The washed microspheres were then lyophilized to form SerMA hydrogel microspheres. The morphology of the hydrogel microspheres were viewed by the light microscopy (Nikon, Japan) and the scanning electron microscopy (SEM) (Hitachi, Japan) after pretreatment of the microspheres in an ion sputtering apparatus.

Gelatin methacryloyl (GelMA) hydrogel microspheres (7.5% w/v) were prepared in the same way as SerMA hydrogel microspheres [34]. To assay the adhence of microspheres to the tissues, 250 µL SerMA and GelMA hydrogel microspheres were injected at the same rate into the injured porcine articular cartilage, and the images and videos of the gross observation were taken.

### Synthesis and characterization of LFDCS

Chs and SerMA (25% w/v) were dissolved in LAP and the hydrogel microspheres were prepared in the same way as SerMA hydrogel microspheres. Chs/SerMA hydrogel microspheres were incubated with Suflo-SMCC for 2 h at 4 °C to form stable amide bonds, and subsequently washed three times with double distilled water, namely activated microspheres [35]. DPG dissolved in double distilled water was soaked with the activated microspheres for 2 h to react with the sulfhydryl group of maleimide. The treated microspheres were dialyzed in an 8,000 kDa dialysis bag for 24 h to form DPG@Chs/SerMA hydrogel microspheres. The prepared liposomes and microspheres were incubated for 12 h and then freeze-dried to form the LFDCS. The morphology of the composite microspheres was photographed by a SEM.

78c@Lipo-FA-DPG@SerMA hydrogel microspheres (LFDS) were prepared in the same way as LFDCS.

To investigate the structure of the material, LFDCS was milled into a powder and examined using a fourier transform infrared (FTIR) spectrometer (Mettler Toledo, USA).

### Friction test

The lubrication properties of SerMA, LFDS, and LFDCS were measured using a general-purpose universal testing machine (UMT-4, Bruker, Germany) under loads of 10 N and 15 N [36]. All tests simulated the physiological movement of the knee joint by mutual friction between an upper 8 mm polyethylene ball (modulus of elasticity: 1.1 Gpa, Poisson’s ratio: 0.42) and a lower Ti6AI4V (modulus of elasticity: 113.8 Gpa, Poisson’s ratio: 0.34) and the sample. The test mode was linear reciprocating motion. The friction stroke was 4 mm, and the amplitude and frequency were 4 mm and 1 Hz, respectively [31]. Each group of microspheres was prepared as a 10 mg/mL PBS and subjected to the friction tests. Each test lasted 800 s.

### Drug encapsulation and liposome responsive release

After centrifugation of 1 mL the liposome solution at 10,000 g for 30 min, the supernatant was aspirated and mixed with an equal amount of dimethyl sulfoxide, and the 78c content was measured at 276 nm with a spectrophotometer (Thermo, USA).

Encapsulation efficiency = Encapsulated drug (Wt)/Total drug (Wt) × 100%.

For the responsive release of liposomes, DiO-stained liposomes were self-assembled with the Lipo-FA-DPG@Chs/SerMA microspheres. MMP9 (20 µg/mL) was added to the experimental group, and the control group were without MMP9. The supernatant was aspirated after 12 h and the images were taken with an inverted fluorescence microscope (Zeiss, Germany).

### In vitro release profile of Chs

Ten mL LFDCS suspension was wrapped in an 8,000 kDa dialysis bag, placed in 50 mL PBS. The Chs content in the liquid at each time (12 h, 1 d, 3 d, 5 d, 7 d, and 14 d) was assessed by high-performance liquid chromatography (HPLC) (Agilent, USA).

### Evaluation of the degradation of LFDCS in vitro

For evaluate th degradation of LFDCS, the frozen dry weight of the microspheres (W_1_) was recorded. Then, the microspheres were placed in 5 mL PBS and incubated at 37 °C. The microspheres were removed from PBS at each time point, and their frozen dry weights (W_2_) were measured. The relative weight was calculated as follows:

Relative weight (%) = W_1_/W_2_ × 100%.

### Isolation and culture of chondrocytes

Cartilage was removed from the male Sprague–Dawley (SD) rats within 24 h of birth [31]. The cartilage was digested with 0.2% Collagenase II for 6 h at 37 °C. Following digestion, chondrocytes were inoculated and cultured in DMEM medium including 10% FBS, and 1% P/S (complete medium). Subsequently, the cells were incubated with 0.2% Collagenase II for 6 h at incubator. Toluidine blue staining was used to identify the chondrocytes. The cells were cultured to the third passage for further use.

For all functional experiments involving chondrocytes, 10 ng/mL IL-1β was added to mimic the inflammatory environment of OA.

### Biocompatibility testing of LFDCS

Microspheres from each group were soaked in DMEM medium for 7 d to obtain the leachate of SerMA, LFDS, and LFDCS, respectively. Chondrocytes were seeded in 96-well plates with the leachate from each group. Cell viability was measured on days 1, 2, and 3 according to CCK-8 instructions.

Chondrocytes were seeded in 12-well plates, and the microspheres from each group were added and cultured for 1, 2, or 3 days. Chondrocytes were stained by the calcein/PI kit and viewed by fluorescence microscope (Zeiss, Germany).

### Macrophage polarization detected by flow cytometry

Complete medium containing 100 ng/mL LPS was used as the induction medium to polarize the macrophages towards M1. First, BMMs were seeded at 1 × 10^5^ in 12-well plates, and then, each group of leachate and induction medium was added to 12-well plates in the incubator for 24 h. BMMs were collected and stained with 0.3 µL CD11b-eFluor 450, 0.3 µL CD86-PE-Cy7, and 0.25 µL CD206-FITC for 30 min. Flow cytometry (Thermo, USA) was conducted to assess the percentage of cell surface markers.

### qRT-PCR

The total RNA of BMMs or chondrocytes cultured in the leachate of each group for 1 d was extracted by TRIzol, and the concentration of total RNA was determined by NanoDrop2000 (Thermo, USA). One µg RNA was transcribed to cDNA using a Script RT kit. SYBR Green was utilized to quantify genetic expressions. The primer sequences for genes can be found in Supplementary Table 1. *GAPDH* was applied for the internal control gene, and the 2^ΔΔCT^ method was used for quantifying gene expression levels.

### Immunofluorescence (IF)

Chondrocytes cultured in the leachate of each group for 3 days were fixed, permeabilized, and sealed. Chondrocytes were treated with a primary antibody solution targeting ACAN and MMP13 overnight. The following day, the cells were treated with a secondary antibody solution for 1 h. Finally, chondrocytes were stained with Phalloidin and DAPI for 10 min and photographed using an inverted fluorescence microscope.

### RNA sequence

Total RNA from chondrocytes treated as the controls (factor-free), and with IL-1β (chondrocytes treated with IL-1β), SerMA (chondrocytes treated with IL-1β and the leachate of SerMA hydrogel microspheres) and LFDCS (chondrocytes treated with IL-1β and the leachate of LFDCS) were collected and analyzed for quality by a bioanalyzer (Agilent Technologies, USA) for quality assessment. Transcriptome sequencing and analyses were performed by Oe Biotech (Shanghai, China).

### ELISA

Chondrocytes were seeded at 5 × 10^4^ in 6-well plates and the leachate of each group were added. After 24 h, the concentration of IL-6 and TNF-α was measured by ELISA kits.

### Treatment of OA in rats

All the experimental animals were housed in a pathogen-free animal facility at the Soochow University Animal Experiment Center (NO. SUDA20230625A04).

After anesthetizing 12-week male SD rats with 2% sodium pentobarbital (50 mg/kg), the rats underwent anterior-cruciate-ligament-transection (ACLT) surgery [31]. In the control group, the knee joint was exposed only. Four weeks after surgery, SD rats were divided at random with four groups according to the treatment: OA + PBS: intra-articular cavity injection of 50 µL PBS; OA + SerMA: 50 µL SerMA microspheres; OA + LFDS: 50 µL LFDS; and OA + LFDCS: 50 µL LFDCS. After another 4 weeks, the SD rats were euthanized, and rat knee joints and viscera were collected.

### Radiographic measurement of the knee joints

After 4 weeks, the SD rats were anesthetized. Magnetic resonance imaging (MRI) (GE Healthcare, USA), X-ray (Faxitron X-ray, USA), and micro-computed tomography (µCT) (Bruker, Germany) were performed to scan the knee joints of SD rats. The width of the knee joint gap was measured using X-ray images. Data reconstruction and calculation of osteophyte volume were performed using NRecon and CTAn software (Bruker).

Regarding rat knee live imaging, LFDCS was modified by Cy5.5 (Duofluor, China). Intra-articular injections of Cy5.5-modified LFDCS were used to detect fluorescence intensity at the rat joints on days 1, 4, 7, and 14 using an in vivo imaging system (AniView 600, China).

### Histochemistry of the knee joints

Rat knee joints were handled as previously described [37]. The paraffin blocks were then cut into 6-µm-thick paraffin sections by microtome (Leica, Germany). The prepared sections were subjected to H&E, Safranin O, and IHC staining (for ACAN and MMP13 and INOS), respectively. Quantitative statistical analysis was performed using the ImageJ software (NIH, USA).

### Statistical analysis

Data were analyzed by GraphPad Prism 8.3.0 software (GraphPad, USA) and expressed as mean ± standard deviation. A paired *t*-test was employed to compare two groups, and a one-way analysis of variance was employed for comparisons involving more than two groups. *P* < 0.05 was considered a significant difference.

## Results and discussion

### Characterization of 78c@Lipo-FA

As shown in Scheme 1, 78c@Lipo-FA was prepared by adding DSPE-PEG2k-FA and 78c proportionally using a thin-film dispersion method. Chs/SerMA hydrogel microspheres were prepared by microfluidics, and the MMP9-responsive peptide DPG was covalently attached to the Chs/SerMA hydrogel microspheres by maleimide and sulfhydryl reactions. Finally, 78c@Lipo-FA, as the secondary structure of microspheres, was prepared to link with DPG@Chs/SerMA hydrogel microspheres to form LFDCS (Scheme 1A).

78c, as a CD38 inhibitor, not only inhibits macrophage function by suppressing CD38 signaling in M1 macrophages, but also exhibits nontoxicity to M2 macrophages, indicating an excellent therapeutic prospect for targeting inflammation in OA [30]. Consistent with previous findings, we found that the M1 macrophage population was significantly lower in LPS + 78c group ( 51.8%) as compared to the LPS group (77.4%) (Figure S1A) [30]. This conclusion was supported by quantitative real-time PCR (qRT-PCR) results for the gene expression of *Tnf-α*, *Il-1β*, and *Inos* (Figure S1B). However, 78c is a fat-soluble compound with low solubility in aqueous solution and is quickly cleared by the synovial fluid, severely reducing the intra-articular drug delivery efficiency [38]. As a drug delivery system, liposomes exhibit the ability to enhance drug loading through the lipophilic properties of phospholipids, thereby promoting the effective uptake of drugs by cells; consequently, they have provided excellent drug delivery in the clinical setting [39].

The folic acid receptor (FAR) family encompasses four members, namely FARα, β, γ, and δ [40]. As a receptor widely expressed upon M1 macrophages, FAR is essentially absent in M0, M2 macrophages, and normal cells [41]. Furthermore, large numbers of FAR-positive macrophages are present in OA [42]. Therefore, we used FA as a ligand to modify liposomes (Lipo-FA) to effectively target M1 macrophages, which did not affect the remaining cells.

**Fig. 1:**
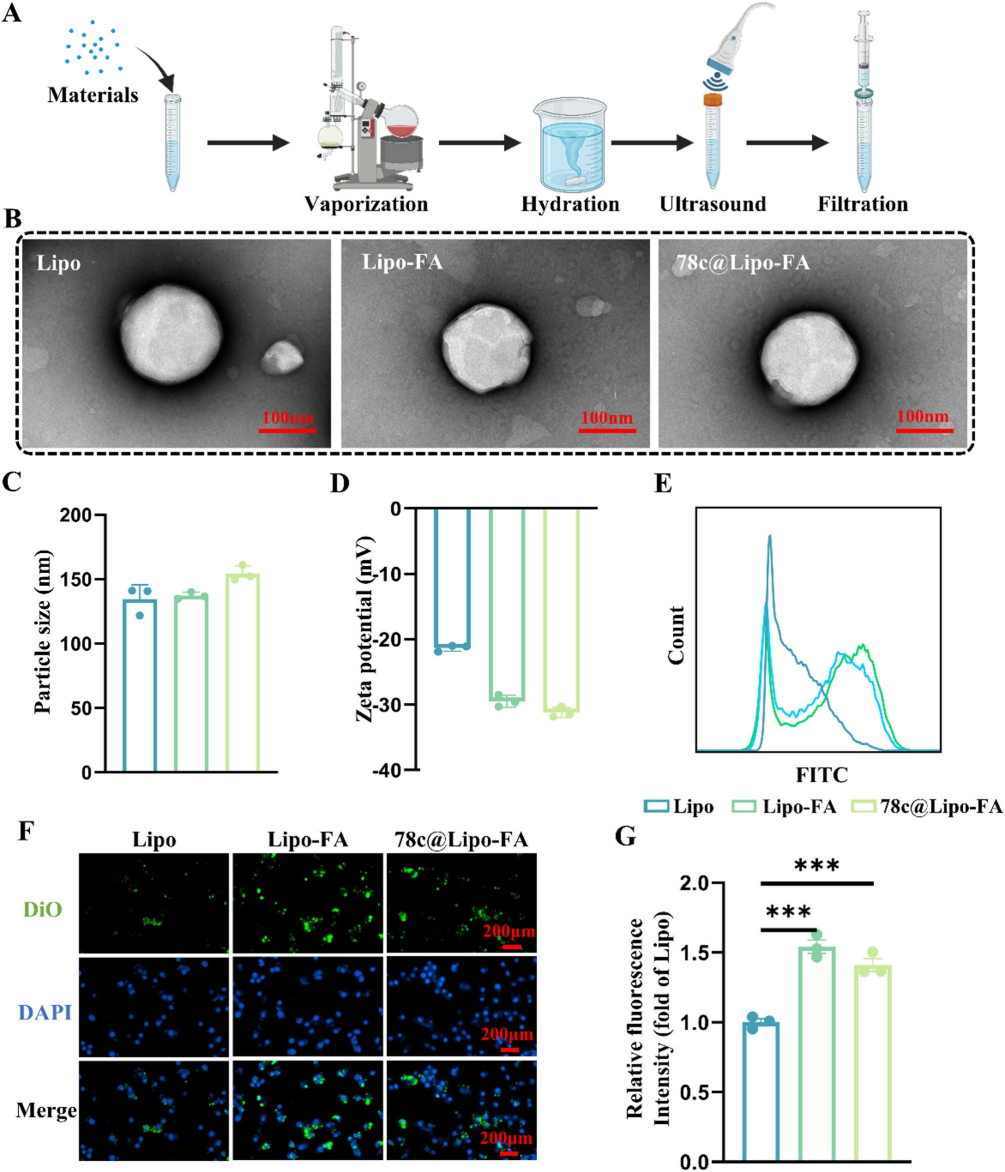
Characterization of 78c@Lipo-FA. (**A**) Scheme diagram of the preparation process of Lipo-FA; materials including 78c, HPSC, cholesterol, octadecylamine, and DSPE-PEG2k-FA. (**B**) TEM of liposomes. (**C**) Particle size of liposomes (*n* = 3). (**D**) Zeta potential of liposomes (*n* = 3). (**E**) Representative panels of the uptaking-liposomes in the BMMs assayed by flow cytometry. (**F**) Representative images of the uptaking-liposomes in the BMMs observed by the fluorescence microscope (After BMMs were incubated for 4 h). (**G**) Relative fluorescence intensity of the uptake of liposomes by BMMs (*n* = 3). ***p < 0.001

In the previous study, liposomes were preparation by thin-film dispersion method. As illustrated in Fig. 1A, liposome films were prepared using a rotary evaporator after mixing the materials in a centrifuge tube. 78c@Lipo-FA was prepared by stirring, ultrasonicating, and extruding the liposome suspension. The TEM results revealed that the liposomes had a uniform spherical appearance (Fig. 1B). The diameters of Lipo, Lipo-FA, and 78c@Lipo-FA were 134.53 ± 11.03 nm, 137.23 ± 2.67 nm, and 154 ± 5.97 nm, respectively (Fig. 1C), and exhibited well dispersion (Figure S1C). The zeta potentials of Lipo, Lipo-FA, and 78c@Lipo-FA were  -21.3 ± 0.49 mV, -46.2 ± 0.95 mV, and  -31.2 ± 0.79 mV, respectively (Fig. 1D, Figure S1D). Interestingly, we found that after FA modification, the zeta potential of the liposomes increased approximately 2-fold, which may be due to the anchoring of the negatively charged functional groups present in the DSPE-PEG2k-FA band to the liposomes [43]. Furthermore, the Lipo-FA encapsulation of 78c was approximately 74% (Figure S2).

To test the targeting of Lipo-FA, flow cytometry assay was employed to assess the uptake of liposomes by BMMs. The results revealed that, compared to Lipo alone, Lipo-FA and 78c@Lipo-FA were taken more by M1 macrophages and the loading of 78c had no significant effect on the targeting of liposomes (Fig. 1E), suggesting that the modification of FA increase the uptake of liposomes. Observed by a fluorescence microscope, as depicted in Fig. 1F and G, M1 macrophages displayed a higher uptake of Lipo-FA and 78c@Lipo-FA than the Lipo group, and the fluorescence intensities of Lipo-FA and 78c@Lipo-FA were 1.5 times and 1.4 times higher than those of the Lipo group, further confirming the targeting of Lipo-FA. In sum, we designed FA-modified liposomes capable of tracking and delivering drugs to M1 macrophages, thus enhancing the liposome delivery efficiency [44].

### Characterization and lubrication properties of LFDCS

SerMA hydrogel exhibits different characteristics at different concentrations [45, 46]. To determine the suitable concentration of SerMA to prepare the microspheres, we examined the G’ and G’’ concentrations of the SerMA solutions during the curing process with a rheometer. As shown in Figure S3, we found that different concentrations of solutions fused well after photo-crosslinking, and 25% w/v SerMA had higher viscosity than the other groups. Therefore, 25% w/v SerMA was selected for subsequent experiments.

**Fig. 2:**
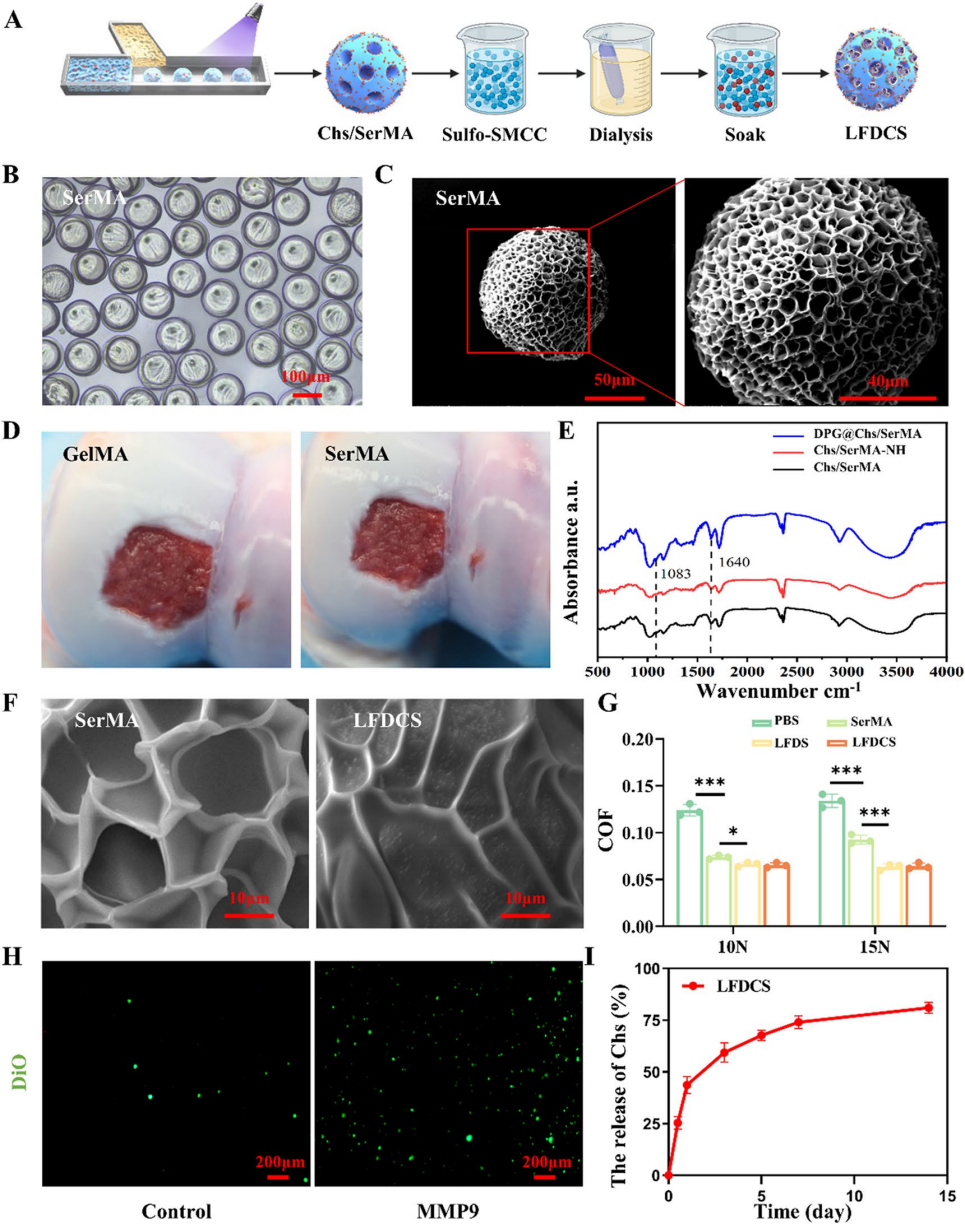
Characterization of LFDCS. (**A**) Scheme diagram of the preparation of LFDCS. (**B**) The morphology of SerMA hydrogel microspheres observed by the light microscope. (**C**) The representative SEM images of SerMA hydrogel microspheres. (**D**) The gross observation of GelMA and SerMA hydrogel microspheres adhension to porcine cartilage. (**E**) FTIR of DPG@Chs/SerMA, Chs/SerMA-NH, and Chs/SerMA. (**F**) The representative SEM image of SerMA and LFDCS. (**G**) COF histograms of PBS, SerMA, LFDS, and LFDCS (*n* = 3). (**H**) The responsive release of liposomes in LFDCS observed by the fluorescence microscope. (**I**) Release of Chs in LFDCS (*n* = 3). *p < 0.05, *** *p* < 0.001

Chs/SerMA hydrogel microspheres were fabricated with a microfluidic device, and the amino groups on the Chs/SerMA hydrogel microspheres were activated by Suflo-SMCC. Subsequently, DPG was covalently attached to the activated Chs/SerMA hydrogel microspheres. After purification by dialysis, 78c@Lipo-FA was bound to DPG@Chs/SerMA hydrogel microspheres *via* DSPE segments embedded in the hydrophobic region of liposomes (Fig. 2A). SerMA was selected as the carrier because of the traits: firstly, sericin contains various reactive groups sensitive to chemical modifications; secondly, sericin possesses high adhesion properties [10, 12, 47]. After demonstrating the excellent physical adhesion properties of 25% w/v SerMA, the microstructure of the microspheres was studied. Fig. 2B showed that the SerMA hydrogel microspheres displayed excellent spherical shape and homogeneity. Scanning electron microscopy (SEM) revealed that SerMA hydrogel microspheres had a porous structure with a diameter of about 100 μm (Fig. 2C).

Currently, microsphere preparation materials such as Chs methacrylate, HA methacrylate, and GelMA, which are commonly employed in the treatment of OA, require chemical modifications to enhance their adhesion properties for cartilage tissue engineering because of their inherently weak adhesion ability [3, 4]. To investigate the impact of SerMA hydrogel microspheres on cartilage adhesion, GelMA hydrogel microspheres were applied as the control group [48]. The results indicated that the microspheres of 25% w/v SerMA hydrogel microspheres exhibited superior adhesion to cartilage and defect sites compared to the microspheres of 7.5% w/v GelMA hydrogel microspheres (Fig. 2D, Supplementary Video 1). Thus, SerMA-based microspheres could effectively adhere to the cartilage surface without additional modification.

FTIR spectroscopy and SEM were performed to confirm the binding of 78c@Lipo-FA to SerMA hydrogel microspheres. The results of FTIR spectroscopy indicated the presence of amide and ether bands at 1,680 cm^− 1^ and 1,083 cm^− 1^ in DPG@Chs/SerMA compared to Chs/SerMA and activated Chs/SerMA. (Chs/SerMA-NH) (Fig. 2E). We also observed the surface morphologies of SerMA, LFDS, and LFDCS by SEM. As shown in Fig. 2F and Fig. S4, some dispersed liposome microreservoirs were observed on the surfaces of LFDS and LFDCS, whereas they were absent in Chs/SerMA or SerMA hydrogel microspheres, suggesting that Lipo-FA are attached to the microspheres.

To explore the lubrication properties of the microspheres, SerMA, LFDS, and LFDCS were placed under 10 N and 15 N loads in the universal material testers, respectively. The results showed that the coefficients of friction (COF) of SerMA, LFDS, and LFDCS were reduced compared with PBS, with the COF values of LFDS and LFDCS decreasing by approximately 49% and 48%, respectively (Fig. 2G, Fig. S5). Owing to the high viscosity and rolling characteristics, SerMA hydrogel microspheres can enhance lubrication by acting as ball bearings [49]. In addition, the results in Fig. 2G revealed that the lubricating properties of LFDS and LFDCS were superior to those of SerMA, indicating that the hydrated synovial membrane provided by liposomes could also reduce the damage to the cartilage by mechanical friction and further synergistically reduce the COF of the interface [36].

Systemic or local injection of targeted liposomes often results in unsatisfactory therapeutic effects because of their short half-lives and limited distances of action [50]. Therefore, it is imperative to establish a liposome preservation reservoir that can preserve liposomes and is programmed to control their release [51]. Abnormal inflammatory states and cartilage friction increase the secretion of IL-6, prostaglandin E2, and MMP3, MMP9, and MMP13 within the joints [52]. In this study, LFDCS were designed as a conserved bank of 78c@Lipo-FA. Upon the presence of MMP9, such as in the microenvironment of the OA and trauma, LFDCS could release 78c@Lipo-FA with DPG cleaved by MMP9 for therapeutic purposes.

As shown in Figure S6, the characteristic peaks of DSPE, PEG2k, and GPLGLAGQC were observed in the nuclear magnetic resonance result, indicating successful synthesis of DPG. Next, DiO dyed-liposomes were observed to study the released 78c@Lipo-FA from LFDCS. In vitro, as expected, more liposomes with green fluorescence were found of LFDCS in the MMP9 environment than in control group without MMP9 (Fig. 2H).

Chs is a vital structural component of the cartilage extracellular matrix (ECM) that promotes chondrocyte anabolism and inhibits chondrocyte catabolism to facilitate cartilage repair [53]. In this study, we prepared SerMA hydrogel microspheres mixed with Chs. First, the concentration of Chs in the LFDCS release solution was determined with HPLC. The results demonstrated that Chs had an initial burst release within 24 h, followed by a gradual plateau, reaching a plateau at 7 d, which reflects approximately 80% encapsulation of Chs (Fig. 2I, Fig. S2). The in vitro degradation profile revealed that LFDCS showed continued biodegradability (Figure S7).

In summary, LFDCS adheres to cartilage and provides lubrication to joints, while also responds to MMP to release liposomes.

### Biocompatibility of LFDCS

**Fig. 3:**
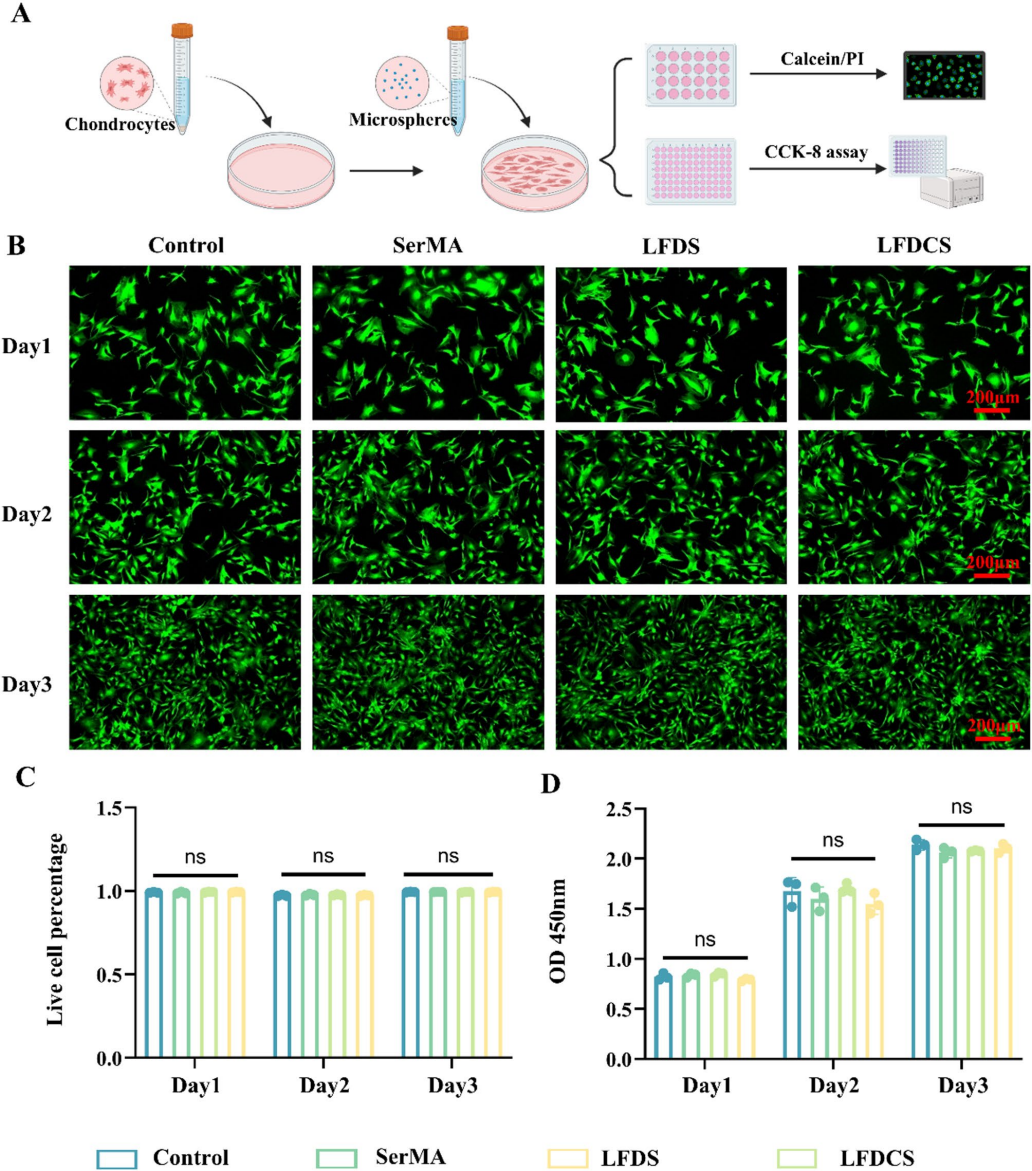
Biocompatibility of LFDCS. (**A**) Scheme diagram of material biocompatibility. (**B**, **C**) Calcein/PI staining and quantitative analysis of chondrocytes on day 1, 2 and 3 (*n* = 3). (**D**) CCK8 assay of chondrocytes viability on day 1, 2 and 3 (*n* = 3). ns, no significant difference

To assess the biocompatibility of the microspheres, rat chondrocytes were successfully isolated evidenced by toluidine blue staining (Figure S8) and cultured in the leachate from each group (SerMA, LFDS, and LFDCS, respectively). Calcein/PI staining and CCK-8 were applied to determine the toxicity of the microspheres to chondrocytes at day 1, 2, and 3, respectively (Fig. 3A). Calcein/PI staining and quantification results demonstrated that there was no significant difference between the groups at the same timepoints (Fig. 3B, C). The CCK-8 assay displayed that the optical density values of the biomaterial group were comparable to those of the control group (Fig. 3D), further confirming that the biocompatibility of LFDCS was good and suitable for applying in vivo.

### **LFDCS inhibited macrophage polarization toward M1 phenotype**

Synovial macrophages are pivotal in generating pro-inflammatory factors (IL-1β and TNF-α) following joint injury [54]. In the synovium of the knee joints of patients with OA, there is a significant activation of M1-mediated inflammation compared with healthy individuals [55]. Despite the secretion of IL-4 and IL-10 by M2 macrophages, these factors are insufficient to counteract inflammatory infiltration, cartilage degradation, and bone regrowth caused by M1 macrophages [22, 56]. Therefore, modulation of intra-articular M1-mediated inflammation is an important strategy for managing OA. As mentioned previously, the inhibition of macrophage CD38 expression effectively inhibited macrophage polarization toward M1 phenotype (Figure S1A).

**Fig. 4:**
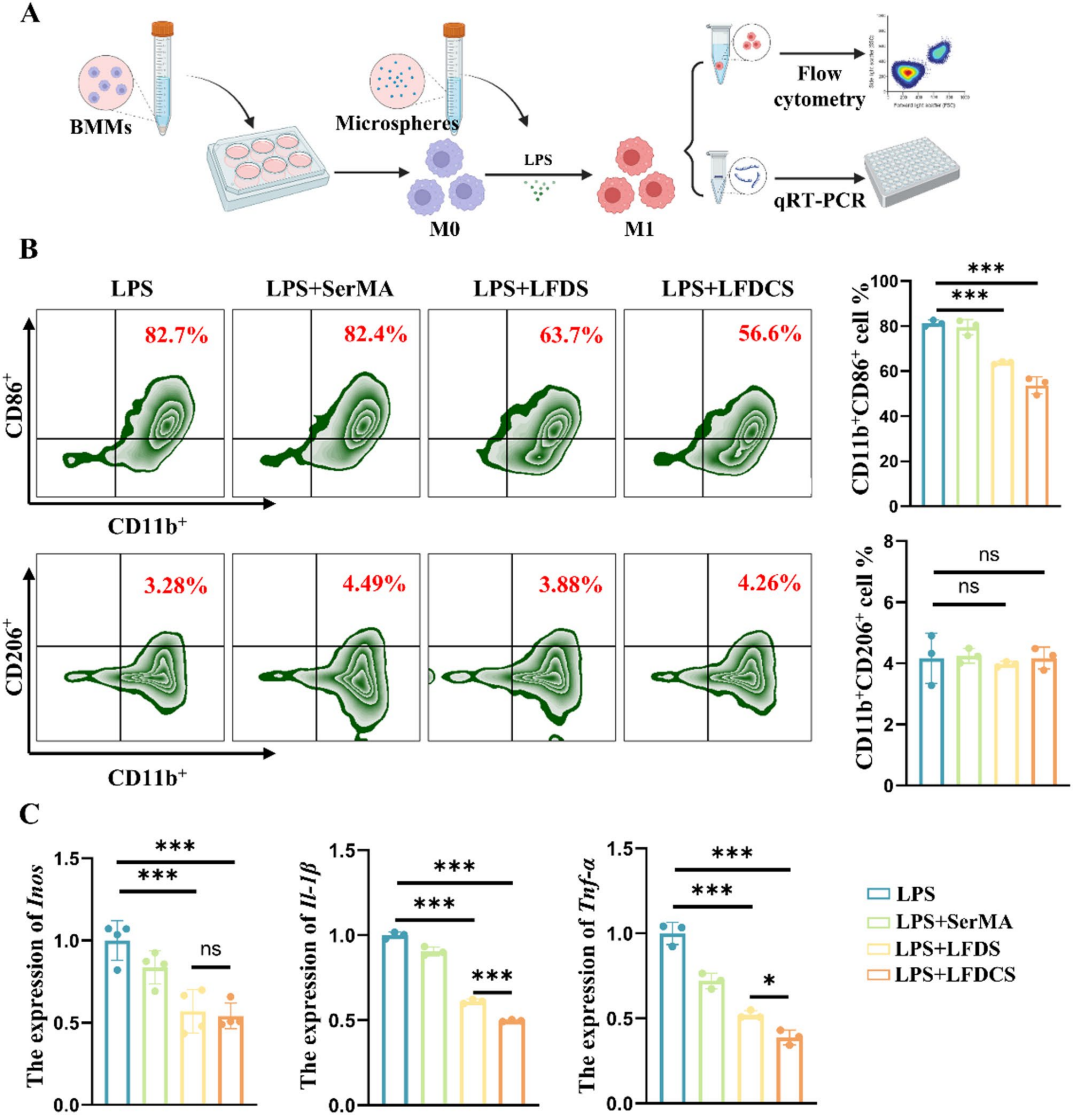
LFDCS inhibited macrophage polarization toward M1 phenotype. (**A**) Scheme diagram of microspheres intervention on macrophage polarization. (**B**) The portion of CD11b^+^CD86^+^ (M1 phenotype) and CD11b^+^CD206^+^ (M2 phenotype) detected by flow cytometry at 24 h (*n* = 3). (**C**) The gene expression of M1 polarization-related genes (*Inos*, *Il-1β* and *Tnf-α*) assayed by qRT-PCR at 24 h (*n* = 3). *p < 0.05, ***p < 0.001, and ns, no significant difference

To examine the influence of LFDCS on macrophage polarization, 100 ng/mL LPS was applied to induce polarization toward M1 phenotype (Fig. 4A). As shown in the Fig. 4B, the proportion of CD11b^+^CD86^+^ (M1 phenotype) BMMs in the LFDS and LFDCS groups decreased by approximately 17.4% and 27.5%, respectively, in comparison to the LPS group, whereas the percentage of CD11b^+^CD206^+^ (M2 phenotype) BMMs was below 6% in both groups.

The expression of *Inos* in the LFDS and LFDCS groups decreased by 0.43 times and 0.46 times, the expression of *Il-1β* by 0.39 times and 0.49 times, and the expression of *Tnf-α* by 0.48 times and 0.62 times, respectively, in comparison to the LPS group (Fig. 4C). Additionally, the proportion of M1 macrophages and the expression of related genes in the LFDCS group were higher than that in the LFDS group, which may be attributable to Chs inhibiting inflammation in M1 macrophages by eliminating ROS [53].

### LFDCS ameliorated IL-1β-induced chondrocyte dysfunction

Inflammation-induced hypertrophic deformation of chondrocytes is an important indicator of OA [57]. Therefore, 10 ng/mL IL-1β was used to elicit inflammation-induced chondrocyte deformation in vitro [58]. ACAN, Col2, and SOX9 are critical factors in chondrocyte regeneration and ECM synthesis, whereas MMP3, MMP13, and ADAMTS4 regulate chondrocyte catabolism [59].

**Fig. 5:**
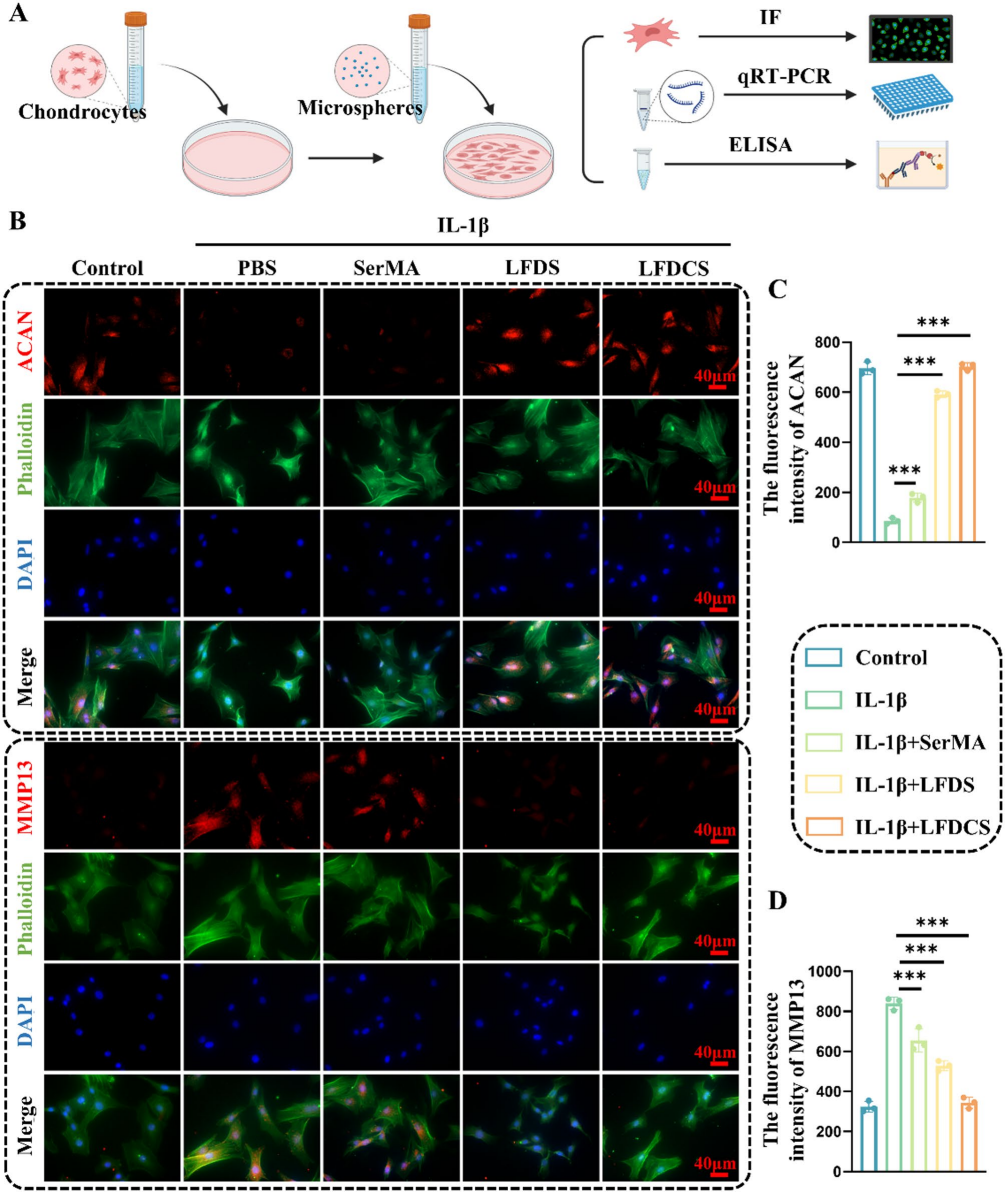
LFDCS up-regulated ACAN and down-regulated MMP13 expression in chondrocytes. (**A**) Scheme diagram of LFDCS intervention in IL-1β-induced chondrocytes. (**B**) The expression of ACAN and MMP13 in the chondrocytes detected by IF. (**C**, **D**) Quantification of ACAN and MMP13 expression in the chondrocytes (*n* = 3). *** *p* < 0.001

Firstly, rat chondrocytes were cultured for 24 h in the leachate from each group, and functional indicators in the chondrocytes were detected by IF staining and qRT-PCR (Fig. 5A). The Fig. 5B demonstrated a gradual increase in ACAN expression and a gradual decrease in MMP13 expression in the chondrocytes in the SerMA, LFDS and LFDCS group, compared with that of the IL-1β group. Quantitative results displayed the expression of ACAN up-regulated 1.06-, 5.84-, and 7.1-fold, while the expression of MMP13 down-regulated by 28%, 59%, and 144%, in the SerMA, LFDS, and LFDCS groups, compared with that in the IL-1β group, respectively (Fig. 5C and D).

In addition, chondrocyte synthesis and catabolism were further assessed by qRT-PCR. In contrast to the results from the IL-1β group, there was a significantly declined gene expression of chondrocyte catabolism-related markers, including *Mmp3* (decreased by 14% and 70%, respectively), *Mmp13* (decreased by 48% and 74%,, respectively), and *Adamts4* (decreased by 52% and 76%, respectively) and a significantly enhanced gene expression of chondrocyte synthesis-related markers including *Sox9* (increased 4.27- and 10.36-fold, respectively), *Col2* (increased 5- and 10.54-fold, respectively), and *Acan* (increased 2.05- and 2.44-fold, respectively) in the LFDS and LFDCS groups. Chs is an important structural component in cartilage and plays an important role in maintaining the cartilage-forming phenotype [53]. The above results indicated that the slow release of Chs in LFDCS group significantly alleviated chondrocyte dysfunction compared to LFDS group.

**Fig. 6:**
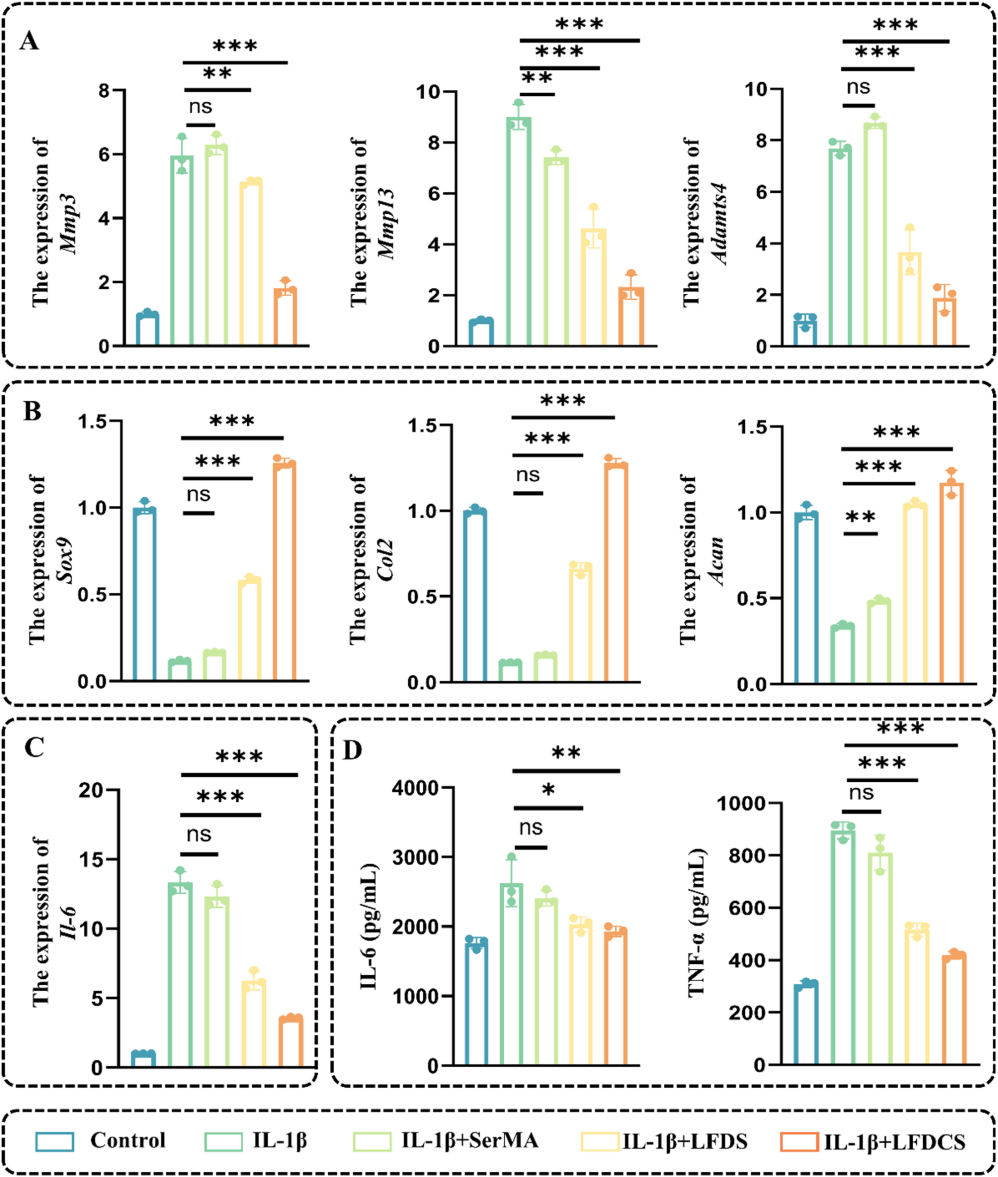
LFDCS ameliorated IL-1β-induced chondrocyte dysfunction. (**A**) The expression of chondrocyte catabolism-related genes (*Mmp3*, *Mmp13* and *Adamts4*) detected by qRT-PCR. (**B**) The expression of chondrocyte anabolism-related genes (*Sox9*, Co*l2* and *Acan*) detected by qRT-PCR. (**C**) The expression of chondrocyte inflammatory gene *Il-6* detected by qRT-PCR. (**D**) IL-6 and TNF-α concentration detected by ELISA. * *p* < 0.05, ** *p* < 0.01, *** *p* < 0.001, and ns, no significant difference

Meanwhile, the inflammatory response was also evaluated. Compared to the IL-1β group, a decrease in the gene expression of *Il-6* (decreased by 53% and 73% in the LFDS and LFDCS groups, respectively) was observed by qRT-PCR (Fig. 6A–C). In the initial phases of OA, chondrocytes reactively secrete TNF-α and IL-6 to exacerbate OA progression [60]. Furthermore, ELISA results revealed that the secretion of IL-6 and TNF-α of chondrocyte were dropped in the LFDS and LFDCS groups, compared to that in the IL-1β groups (Fig. 6D). Interestingly, the LFDS group had the ability to promote cartilage function compared to the SerMA group (Fig. 5B). This may be due to the fact that the 78c released from the leachate of the LFDS group directly acted on the chondrocytes to ameliorate chondrocyte function [29, 30].

### LFDCS attenuated fluid-phase signaling and reduced osteoid formation in the joints of OA rats

**Fig. 7:**
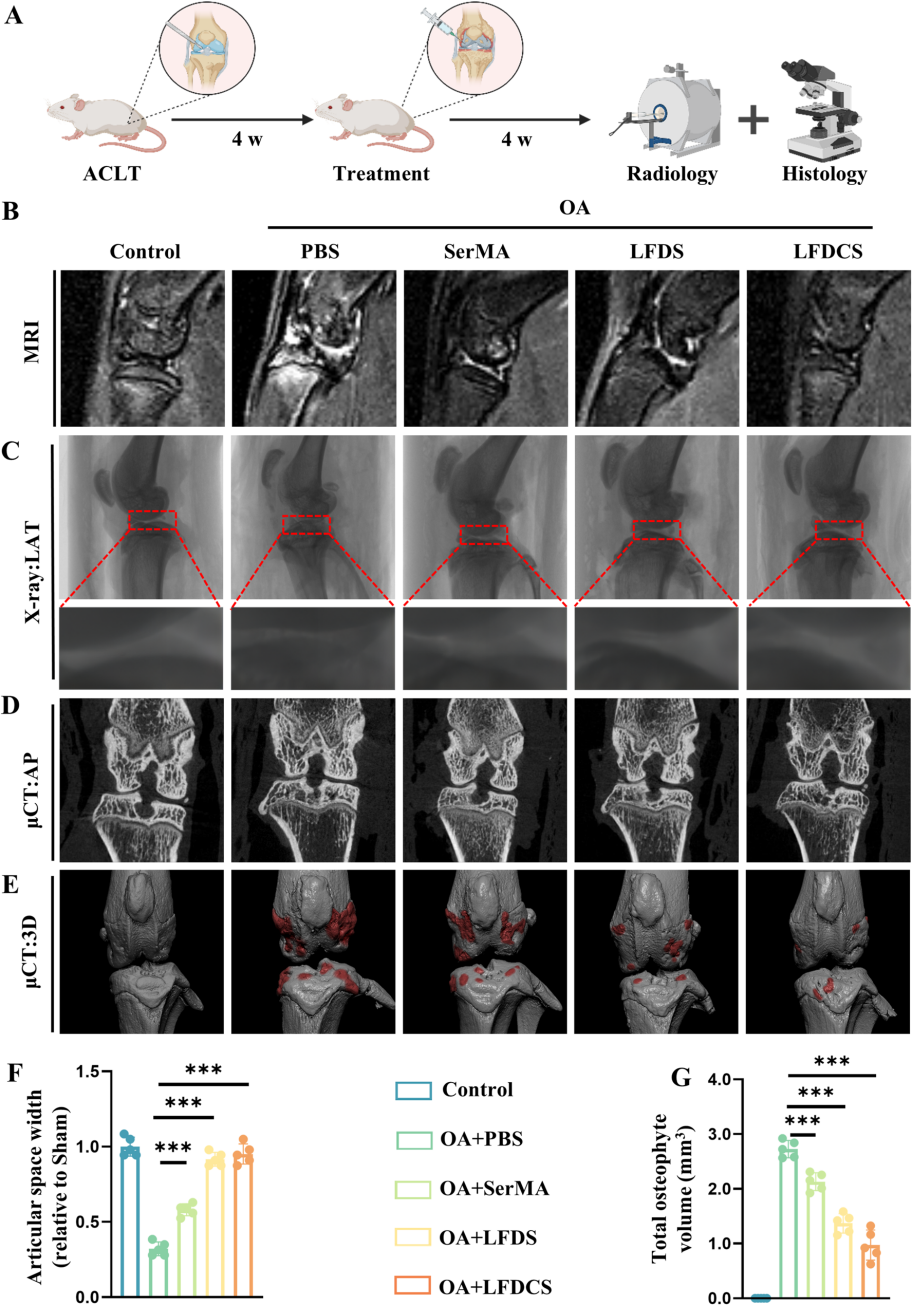
LFDCS reduced fluid-phase signaling and osteoid formation in the joints of OA rats. (**A**) Scheme diagram of LFDCS treatment. (**B**) Edema in the joint cavity detected by MRI scanning. (**C**) Articular surfaces detected by X-ray scanning, LAT: lateral. (**D**) Articular surfaces detected by µCT scanning, AP: anteroposterior. (**E**) 3D reconstruction of the knee joint by µCT scanning. (**F**) X-ray quantification of the medial cartilage gap width in rats (*n* = 5). (**G**) Quantification of total osteophyte in the rat knee joint (*n* = 5). *** *p* < 0.001

In vitro, we verified that LFDCS promoted cartilage adhesion, attenuated M1 macrophage inflammation, and ameliorated chondrocyte dysfunction; therefore, we applied LFDCS in rats with anterior cruciate ligament transection (ACLT) [61]. One month after surgery, rats were randomly divided and injected with PBS and the microspheres (SerMA, LFDS, and LFDCS, respectively) in the articular space and recorded as the PBS, SerMA, LFDS, and LFDCS groups, while the rats with sham surgery were set as the control group. After 4 weeks of treatment, the rats were scanned with MRI and µCT, and their joints were collected for histological staining (Fig. 7A). The in vivo compatibility test revealed that the injection of microspheres had no significant effect on the internal organs in each group (Fig. S9A).

To explore the retention of LFDCS in the joint cavity, Cy5.5 was loaded onto LFDCS, and the change of fluorescence signal in the joint cavity after surgery was observed by an in vivo imaging system. One month after ACLT, with intra-articular injection of Cy5.5-labled LFDCS, the fluorescence signal in the joint cavity was maintained at a relatively stable level at first, and gradually decreased with the gradual degradation of LFDCS. Till to the 14th day, a fluorescence signal was still visible in the joint cavity, indicating that LFDCS have an excellent slow-release capability in the joint cavity (Fig. S9B).

According to the MRI results, the fluid-phase signals in the joint cavities of the rats in the PBS, SerMA, LFDS, and LFDCS groups gradually decreased compared with that of the control group (Fig. 7B). And the fluid-phase signals of LFDCS were similar to that of the control group, which indicated that the administration of LFDCS greatly reduced joint edema. Narrowing of the joint space on radiographs usually indicates OA with progressive cartilage damage [62]. Lateral knee X-rays revealed that the articular space width in the PBS group decreased 2.13-fold compared with the control group, whereas the articular space width in the SerMA group increased 0.81-fold, the LFDS group increased 1.88-fold, and that in the LFDCS group increased 1.97-fold compared with the PBS group (Fig. 7C and F). According to the anteroposterior view of the knee joint, medial subchondral osteosclerosis was significantly more severe in the PBS group than in the control group. However, it was alleviated by being treated with SerMA, LFDS, and LFDCS, compared with that of the PBS group (Fig. 7D).

Intraarticular osteophyte formation is another pathogenic feature of OA [63]. The results of µCT and 3D reconstruction indicated that the degree of intra-articular osteophyte formation was greater in the PBS group than in the control group. However, the degree of osteophyte formation was alleviated in the SerMA, LFDS and LFDCS groups (Fig. 7E, G). According to the results shown in Fig. 7B-E, the improvement of OA by LFDCS was significantly better than that in the LFDS and SerMA groups, which was consistent with the results in the in vitro experiments. It suggested that LFDCS not only prevent further joint damage by reducing joint friction, but also promotes the repair of already damaged cartilage by slowly releasing Chs.

### LFDCS reduced cartilage damage in OA rats

**Fig. 8:**
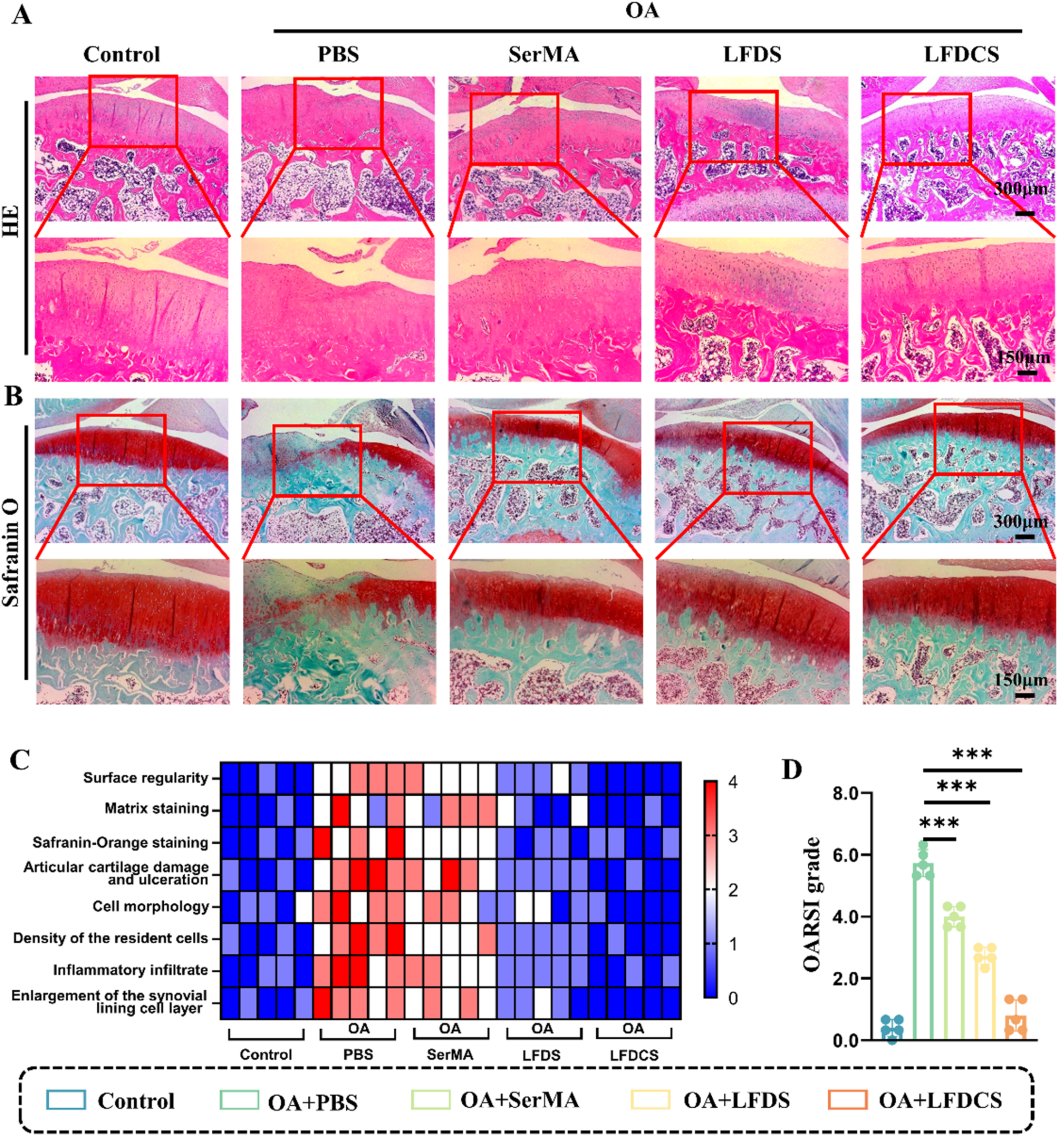
LFDCS attenuated cartilage wear in OA rats. (**A**) H&E staining of articular surface wear in OA rats. (**B**) Safranin O staining of cartilage surface in OA rats. (**C**) Heatmap of variables of histological scoring (*n* = 5). (**D**) OARSI grades of rat joints (*n* = 5). *** *p* < 0.001

For histological analysis of the cartilage, the control group had smooth cartilage surfaces and uniform ECM. The PBS group had severe cartilage defects and abnormal cellular arrangement. In contrast, these impairments were mitigated in the SerMA, LFDS, and LFDCS groups compared with the PBS group (Fig. 8A). Notably, although the degree of cartilage wear was similar in the SerMA and PBS groups, the SerMA group had a more complete cartilage structure and more neatly arranged cells than the PBS group did. Thus, SerMA hydrogel microspheres injection is beneficial for delaying OA progression.

Safranin O staining was performed on rat cartilage sections to differentiate cartilage and bone. More severe cartilage defects and fibrous hyperplasia around the articular surfaces were observed in PBS group than in the control group. Corresponding to the results of radiology and H&E staining, cartilage wear significantly improved in the SerMA, LFDS, and LFDCS group compared with the PBS group (Fig. 8B). Compared to the PBS, SerMA and LFDS groups, the cartilage around the articular surfaces in the LFDCS group was more evenly aligned, with less cartilage wear and less fibroplasia (Fig. 8B). The microstructure of the repaired cartilage was graded by means of a histological score to quantify the degree of cartilage healing. The results demonstrated a gradual increase in cartilage repair scores with treatment in the SerMA, LFDS, and LFDCS groups compared to the PBS group (Fig. 8C). The OARSI scores for the PBS, LFDS, and LFDCS groups were approximately 5.73, 2.73, and 0.8, respectively, confirming that LFDCS can significantly delay OA progression (Fig. 8D).

### LFDCS ameliorated cartilage dysfunction and attenuated synovial inflammation in OA rats

**Fig. 9:**
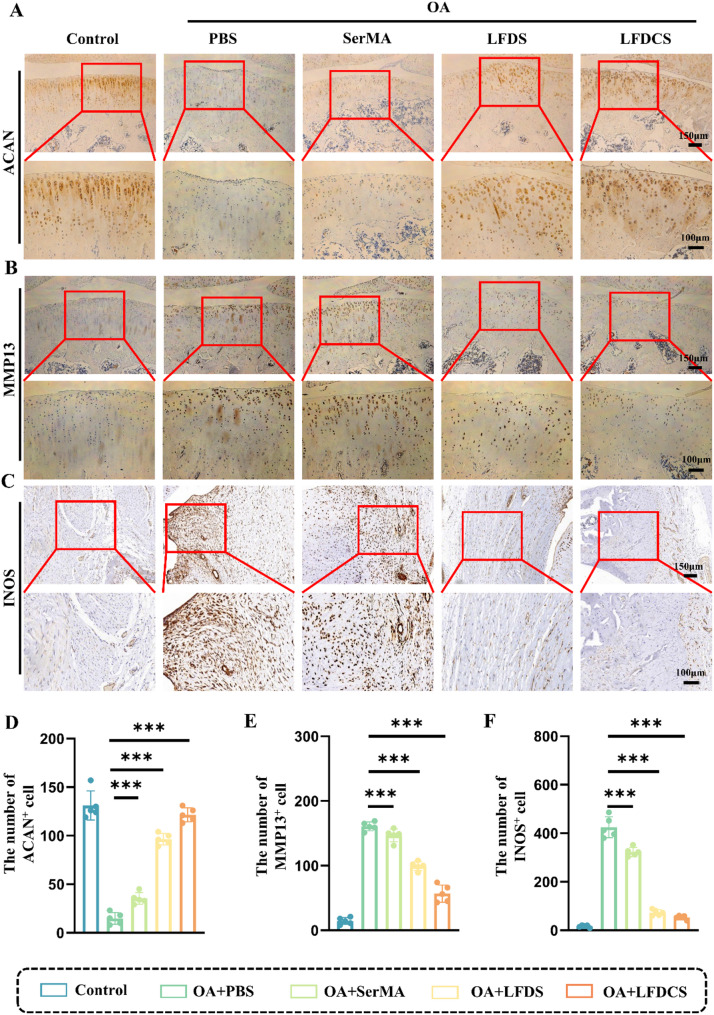
LFDCS improved cartilage dysfunction and alleviated synovial inflammation in OA rats. (**A**-**C**) The expression of ACAN, MMP13 and INOS in rat joints detected by IHC. (D-E) Quantification of the number of ACAN, MMP13 and INOS by IHC (*n* = 5). *** *p* < 0.001

We further analyzed the expression of ACAN and MMP13 on the cartilage surface by IHC analysis. As shown in Fig. 9A, B, D, E, there was a 1.39-fold increase in the expression of ACAN in the SerMA group, a 5.5-fold in the LFDS group, and a 7.21-fold in the LFDCS group, whereas the expression of MMP13 was reduced by 8% in the SerMA group, 38% in the LFDS group, and 65% in the LFDCS group compared to the PBS group. Intraarticular injection of LFDCS substantially promoted the expression of ACAN and suppressed the expression of MMP13 on the joint surface (Fig. 9A, B). This is consistent with our speculation in the in vitro part of the experiment. 78c in the LFDS group mostly acted on M1 macrophages, and the slow release of Chs in LFDCS further ameliorated chondrocyte dysfunction when subjected to extrusion. Interestingly, the higher ACAN and the lower MMP13 in the SerMA group than in the PBS group may be explained by the fact that SerMA hydrogel microspheres act as a bionic bearing to reduce cartilage wear and chondrocyte damage, thereby improving chondrocyte metabolism. In summary, LFDCS can be used as both a biolubricant and a drug delivery vehicle to attenuate cartilage wear and tear, minimize osteophyte, and promote ECM production, thereby alleviating OA progression. In addition, to further verify the flow cytometry results of Fig. 4, we analyzed the expression of INOS at the synovium of rat joints by IHC. As shown in Fig. 9C, F, the expression of INOS was decreased in the LFDS and LFDCS groups compared with the PBS group, which indicated that the intervention of 78c significantly suppressed macrophage inflammation. In sum, it is illustrated that under the high-MMP9 environment of OA, most of the 78c@Lipo-FA released from the LFDCS exerted a role in targeting M1 macrophages, while Chs located within the LFDCS well exerted a role in ameliorating chondrocyte dysfunction.

The pathology of OA is usually related to inadequate vascular perfusion, inflammation, and decreased lubrication [22, 64]. The interaction between recurrent inflammatory responses and cartilage wear and tear in the joints is a significant driver of OA [57]. The novel microsphere drug delivery system we developed ameliorates the two major etiological factors of OA progression (inflammation and increased friction) and also promotes cartilage adhesion to improve drug efficiency. Hyaluronic acid (HA) injection remains a widely utilized clinical intervention for OA symptom alleviation [65]. Although HA therapy restores intraarticular viscoelastic equilibrium, its therapeutic efficacy is confined to transient symptomatic relief and necessitates repeated administration [66]. In contrast, hydrogel microsphere systems demonstrate superior injectability and biocompatibility while serving as a scaffold for chondroprotective drug conjugation, thereby achieving synergistic effects in cartilage lubrication and regeneration [67]. However, current hydrogel microsphere platforms exhibit an inherent compromise between optimal lubrication performance and drug delivery efficiency [68, 69]. In vivo experimental, we revealed that the LFDCS composite microsphere system not only exhibited superior lubrication characteristics but also demonstrated enhanced adhesion to cartilage surfaces, thereby amplifying its reparative efficacy on damaged articular tissue. The development of LFDCS microspheres presented a novel therapeutic strategy for cartilage repair and regeneration in osteoarthritic pathologies, bridging the critical gap between mechanical functionality and biological restoration in current regenerative medicine approaches. However, the preparation of LFDCS is not yet mass-produced, and further research and simplification is still needed. In the future, we will continue to focus on material optimization and clinical translation.

### RNA analysis of chondrocyte improvement by LFDCS

To delve deeply into understanding the mechanism of action of the complex microspheres on chondrocytes, we performed RNA transcriptome sequencing of chondrocytes subjected to different treatments. First, we analyzed the differentially-expressed genes (DEGs) in the IL-1β and control groups. As shown in the Fig. S10A, compared with the control group, IL-1β intervention down-regulated the expression of chondrocyte catabolism and inflammation-related genes *Adamts4*, *Mmp3*, *Mmp9*, and *Il-6*, and up-regulated the expression of *Col2a1* and *Sox9*, which proved that the model of IL-1β-induced chondrocyte dysfunction was successfully established.

**Fig. 10:**
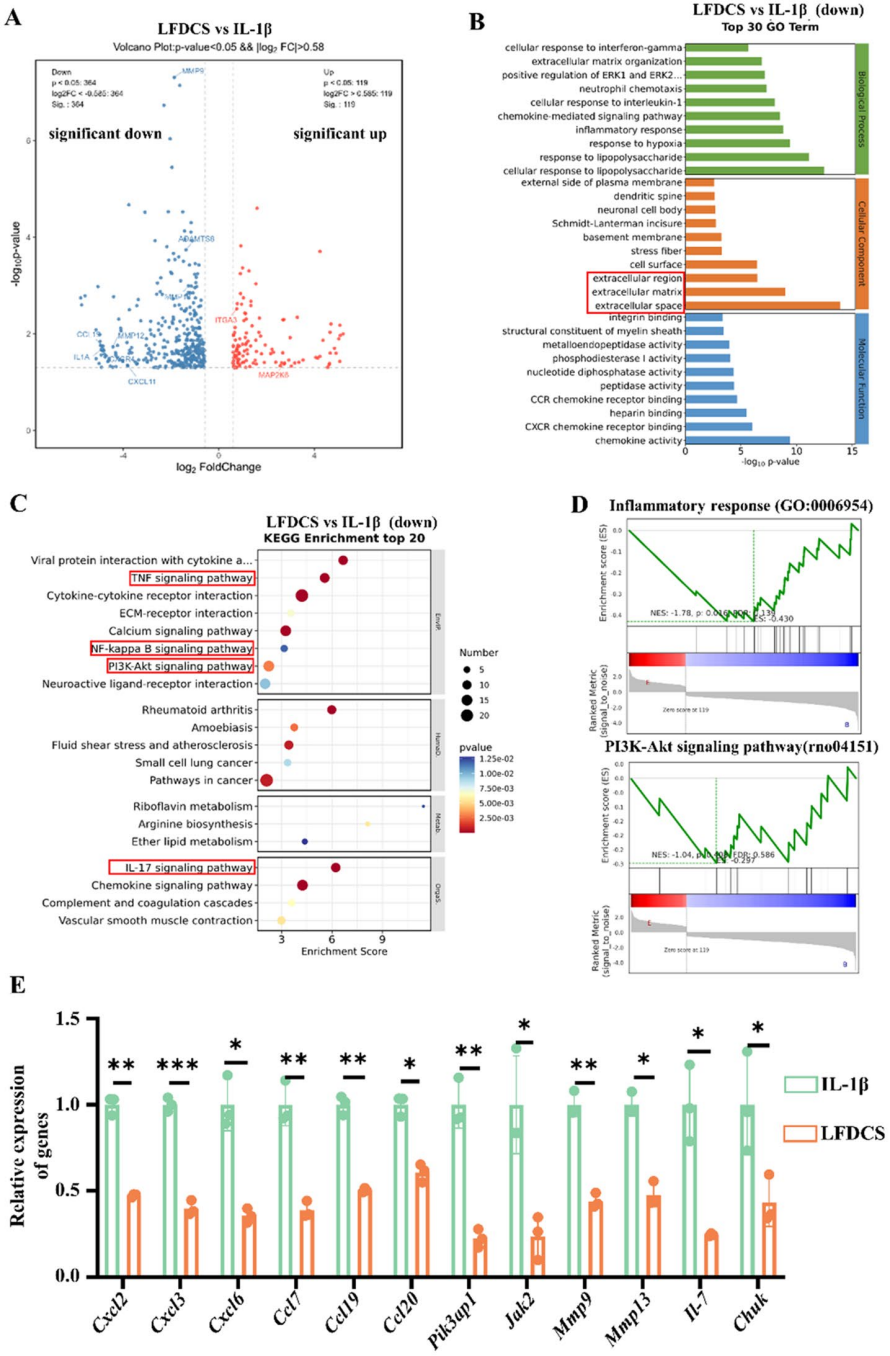
RNA sequencing of the IL-1β-treated chondrocytes with or without LFDCS. **.** (**A**) DEGs between IL-1β and LFDCS groups from volcano map analysis. (**B**) Cellular component of DEGs between IL-1β and LFDCS groups from GO enrichment analysis. (**C**) Signaling pathways of DEGs between IL-1β and LFDCS groups from KEGG enrichment analysis. (**D**) GSEA of inflammatory response and PI3K-Akt signaling pathway gene set in DEGs between IL-1β and LFDCS groups. (**E**) The expression of *Cxcl2*, *Cxcl3*, *Cxcl6*, *Ccl7*, *Ccl19*, *Ccl20*, *Pik3ap1*, *Jak2*, *Mmp9*, *Mmp13*, *Il7*, *Chuk* detected by qRT-PCR (*n* = 3). * *p* < 0.05, ** *p* < 0.01, *** *p* < 0.001. IL-1β: chondrocytes treated with IL-1β; LFDCS: chondrocytes treated with IL-1β and the leachate of LFDCS

Next, by analyzing the DEGs in the IL-1β group (chondrocytes treated with IL-1β) and the LFDCS group (chondrocytes treated with IL-1β and the leachate of LFDCS), 273 down-regulated genes and 73 up-regulated genes were detected (Fig. S10B). The volcano plot results showed down-regulation of pro-inflammatory genes (*Ccl9*,* Cxcl11*, and *Il-1a*), down-regulation of chondrocyte catabolic genes (*Mmp12* and *Mmp13*), and up-regulation of ECM synthesis genes (*Itga3* and *Map2k6*) in the LFDCS group in comparison to the IL-1β group (Fig. 10A). Gene ontology (GO) enrichment analysis displayed that the effects of LFDCS on the cellular components of chondrocytes were mainly focused on the regulation of extracellular regions, ECM, extracellular space, and ECM organization, indicating that LFDCS ameliorated chondrocyte dysfunction by promoting the ECM process (Fig. 10B).

Subsequent analysis by Kyoto Encyclopedia of Genes and Genomes (KEGG) displayed LFDCS regulates ECM synthesis through modulation of the TNF, NF-κB, IL-17, and PI3K/AKT signaling pathways (Fig. 10C). Besides, we performed gene set enrichment analysis (GSEA) of the DEGs. The results indicated that the LFDCS group down-regulated the chondrocyte inflammatory response-related and the PI3K/AKT signaling pathway compared to the IL-1β group (Fig. 10D). PI3K/AKT is an essential signaling pathway involved in various functions that maintain cellular homeostasis [70]. Studies show that down-regulation of the PI3K/AKT signaling pathway can alleviate chondrocyte deformation by activating mitochondrial autophagy [71, 72]. Simultaneously, we verified the DEGs of interest in the RNA sequencing results by qRT-PCR. These results were in accordance with the sequencing analysis (Fig. 10E), which further indicated that LFDCS improved chondrocyte metabolism by regulating the chondrocyte inflammation-associated and the PI3K/AKT signaling pathway.

In addition, chondrocyte function was improved to a greater extent in the SerMA group (chondrocytes treated with IL-1β and the leachate of SerMA hydrogel microspheres) compared to the IL-1β group (Fig. 5 and 6). Moreover, the LFDS exhibited a greater efficacy in enhancing cartilage function than the SerMA did. We performed KEGG enrichment analysis of DEGs from the IL-1β and SerMA groups. The results showed that the SerMA group inhibited chondrocyte inflammatory signaling pathway activity, including the NF-κB and chemokine signaling pathways (Fig. S10C). Moreover, some studies have demonstrated that SerMA may promote cartilage function *via* the ability of silk gum to combat IL-1β-induced chondrocyte dysfunction through scavenging ROS [73]. Our in vivo and in vitro studies displayed that SerMA hydrogel microspheres, a bioactive material, was also favorable to promoting articular cartilage repair. Moreover, inhibition of CD38 expression in chondrocytes ameliorates chondrocyte dysfunction and alleviates OA [29, 30].

However, this study had some limitations. First, we did not explore the responsive release of 78c@Lipo-FA in vivo. Second, we did not explore the mechanics of the LFDCS in detail. Third, the mechanism regarding the responsiveness of LFDCS to the local microenvironment of OA was not elucidated. Therefore, follow-up experiments should focus on the characterization of the material and its specific mechanism of action on chondrocytes and BMMs.

## Conclusion

Inspired by “tapioca pearls”, we successfully fabricated LFDCS, the bionic bearing-inspired lubricating microspheres characterized by M1-phenotype macrophage-targeted immunoregulation physiologically- and physically-coupled. Our experiments demonstrated that the system physically lubricate the cartilage *via* a high adhesion Chs/SerMA hydrogel microsphere, physiologically inhibited M1 macrophage-mediated inflammation *via* responsive release of 78c@Lipo-FA, and promoted chondrocyte function by releasing Chs *via* motor extrusion of Chs/SerMA hydrogel microspheres. Mechanically, the LFDCS ameliorated chondrocyte dysfunction *via* the improvement of chondrocyte metabolism by modulating inflammation and the PI3K/AKT signaling pathway. This novel drug delivery system coupling physical and physiological characters has great potential for application in the clinical OA setting.

## Electronic supplementary material

Below is the link to the electronic supplementary material.

Supplementary Material 1

Supplementary Material 2

## Data Availability

No datasets were generated or analysed during the current study.
