# Supplementary material for: Bionic bearing-inspired lubricating microspheres with Immunomodulatory effects for osteoarthritis therapy
Source: J Nanobiotechnology. 2025 Jun 20;23:457. doi: 10.1186/s12951-025-03544-2 (PMC12180219; doi:10.1186/s12951-025-03544-2)
Supplement: Supplementary file 2 — Supplementary Material 2 [file 12951_2025_3544_MOESM2_ESM.docx]

**Bionic bearing-inspired lubricating microspheres with immunomodulatory effects for osteoarthritis therapy**

*Xu He, Xingzhi Liu, Xingyi Cheng, Can Zhu, Yida Chen, Yi Wang, Yun Zhou, Hao Shen, Huilin Yang, Yong Xu*, Qin Shi*, Junjie Niu**

X. He, X. Cheng, C. Zhu, Y.Chen, Y. Wang, Y. Zhou, H. Shen, Prof. H. Yang, Prof. Y. Xu, Prof. Q. Shi, Prof. J. Niu

Department of Orthopedics, The First Affiliated Hospital of Soochow University, Orthopedic Institute of Soochow University, Medical College of Soochow University, 899 Pinghai Road, Suzhou, Jiangsu 215031, P. R. China

E-mail: [yxu1615@suda.edu.cn](mailto:yxu1615@suda.edu.cn); shiqin@suda.edu.cn; niujunjie1129@suda.edu.cn

Dr. X. Liu

Rui Jin Hospital, Lu Wan Branch, Shanghai Jiao Tong University School of Medicine, NO.149 South Chongqing Road, Shanghai 200000, P. R. China

**Keywords:** Osteoarthritis, Microsphere, Cartilage adhesion, Inflammatory, Sericin methacryloyl, Chondroitin sulfate


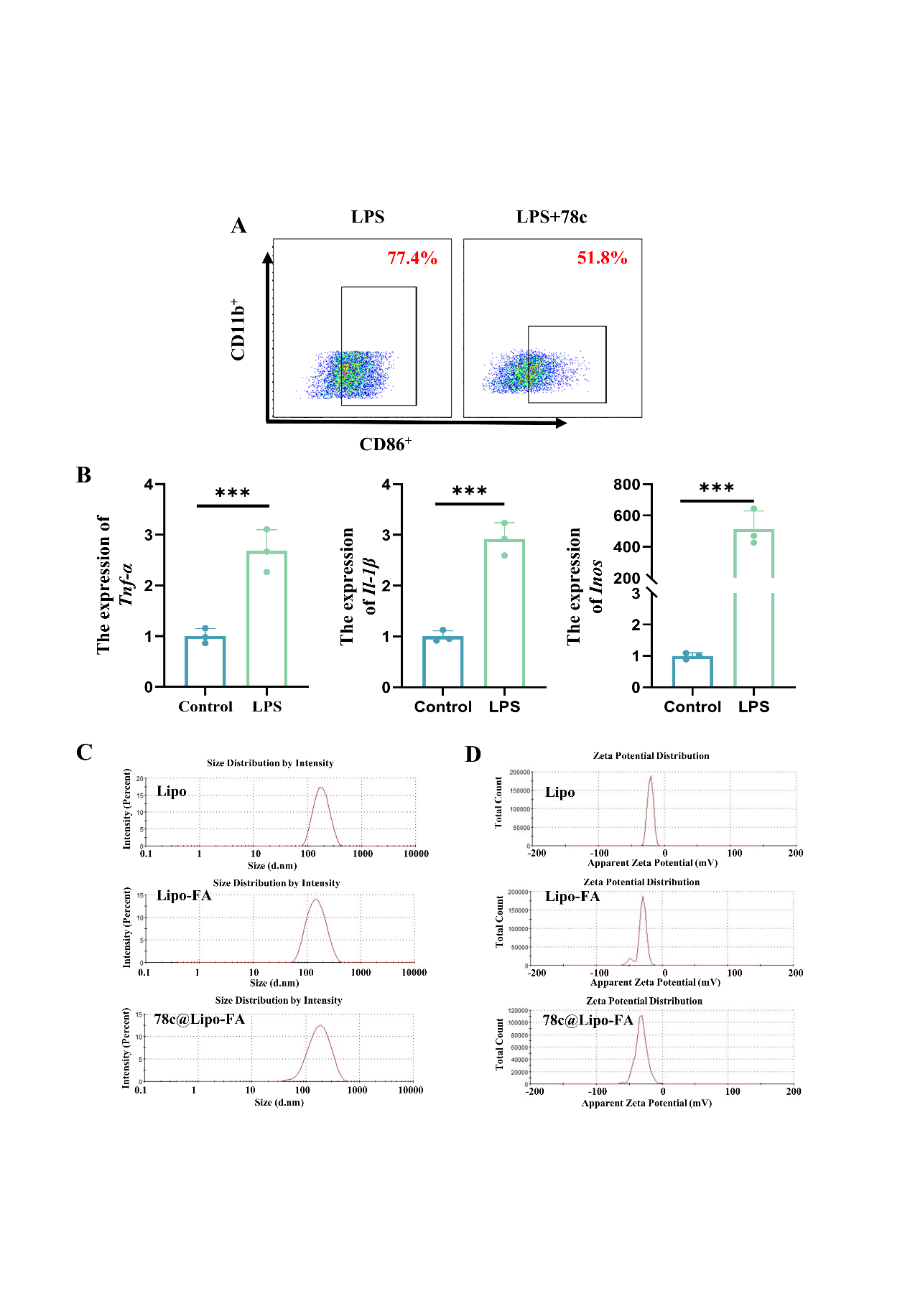


**Supplementary Figure 1.** (A) The portion of CD11b^+^CD86^+^ (M1 phenotype) detected by flow cytometry at 24h. (B) The gene expression of M1 polarization-related genes (*Tnf-α*, *Il-1β*, and *Inos*) assayed by qRT-PCR at 24h (n=3). (C) The particle size of Lipo, Lipo-FA, and 78c@Lipo-FA detected by nanoparticle analyzer. (D) The zeta potential of Lipo, Lipo-FA, and 78c@Lipo-FA detected by nanoparticle analyzer. *** *p* < 0.001.


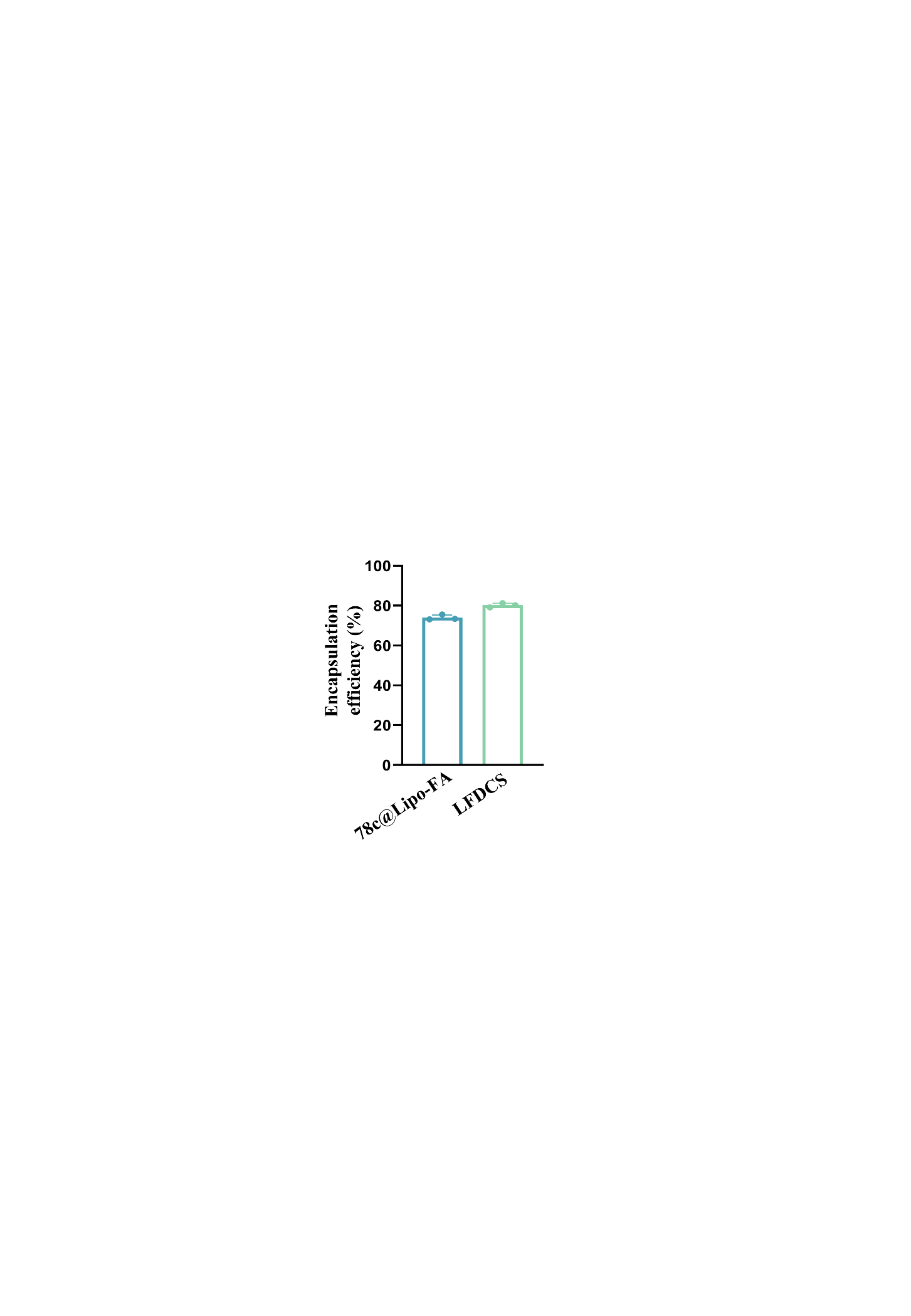


**Supplementary Figure 2.** Encapsulation rate of liposomes and LFDCS (n=3).


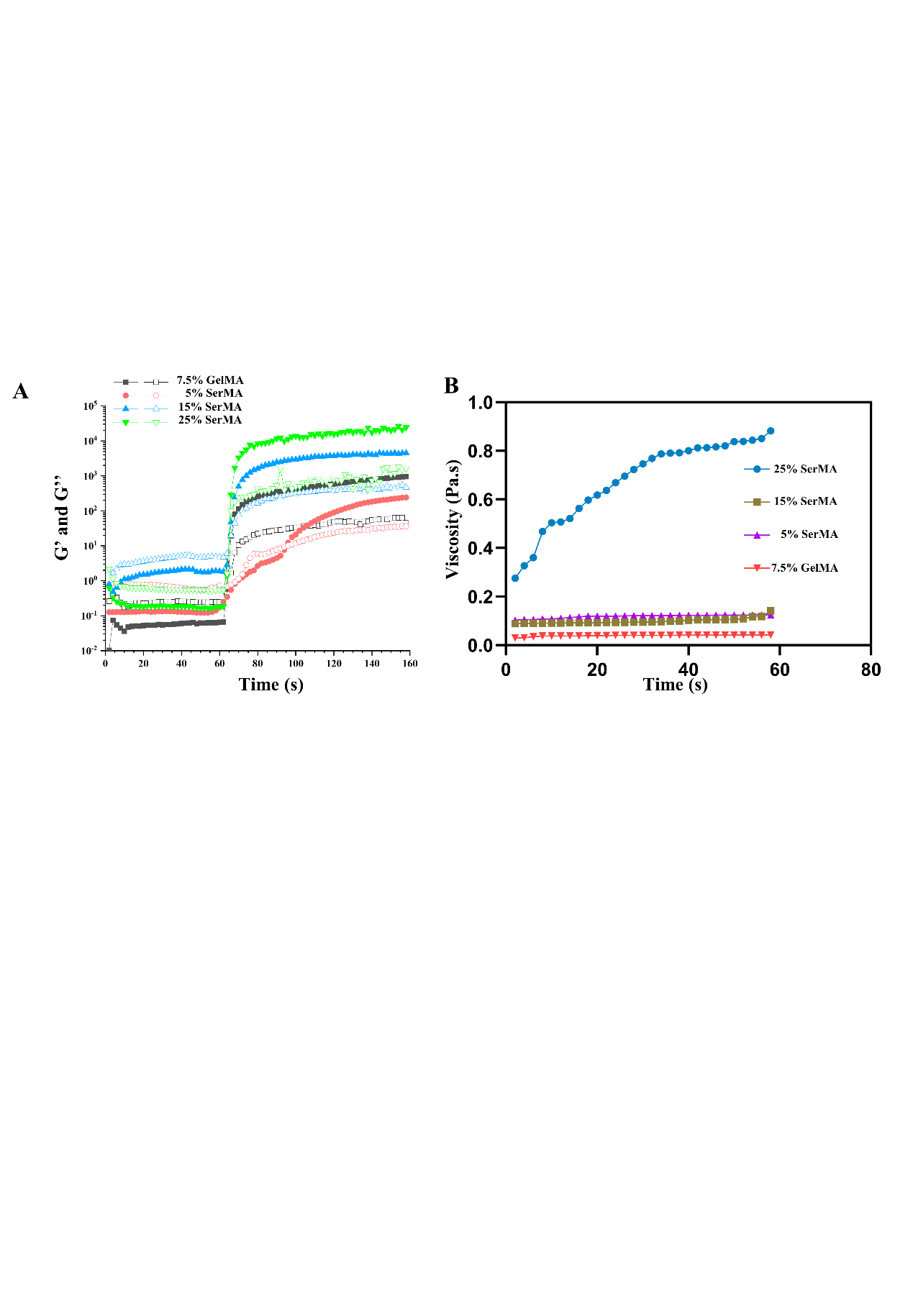


**Supplementary Figure 3.** (A) Hydrodynamic detection of different concentrations of SerMA hydrogel. (B) Viscosity of different concentrations of SerMA hydrogel.


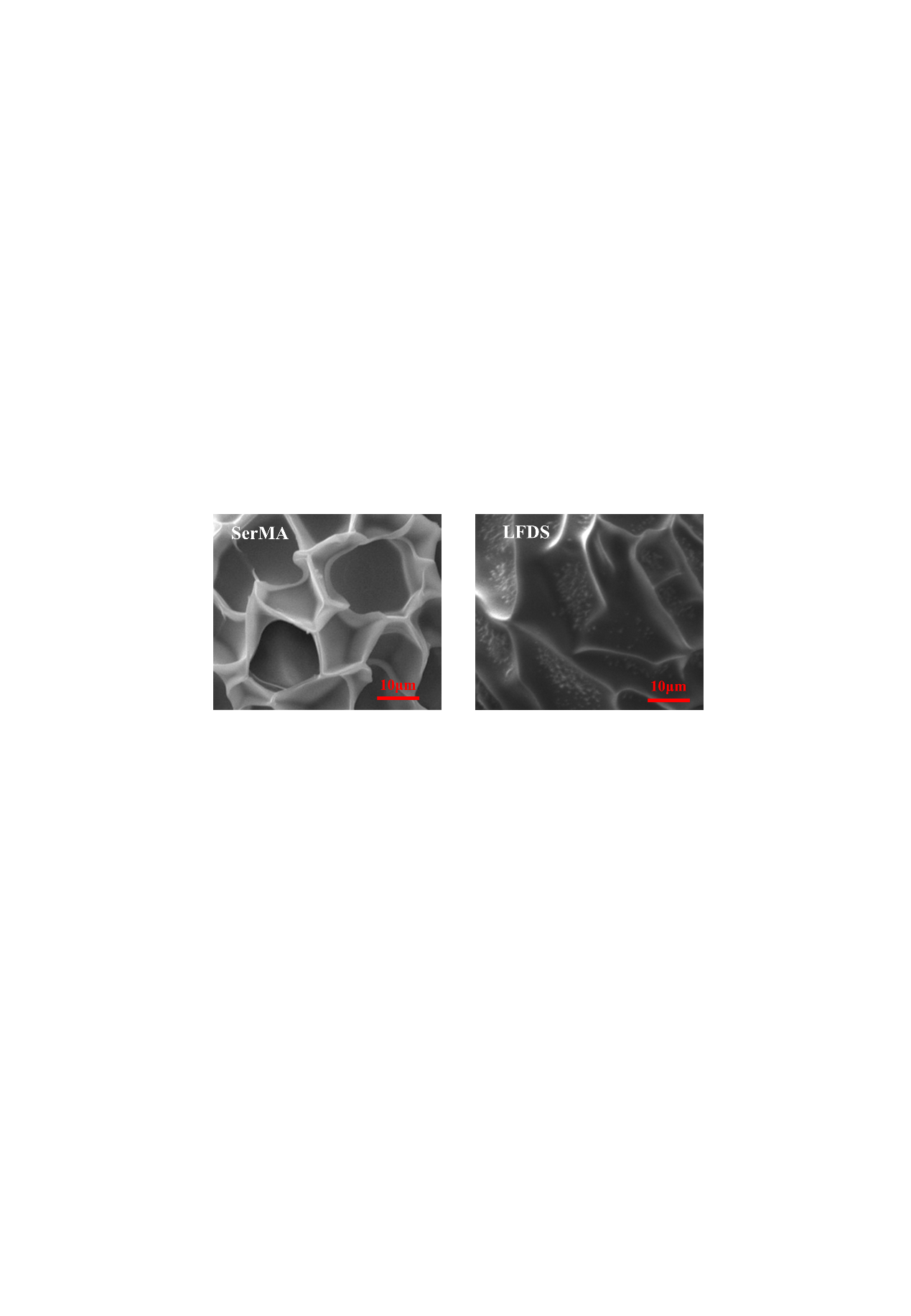


**Supplementary Figure 4.** SEM image of LFDS.


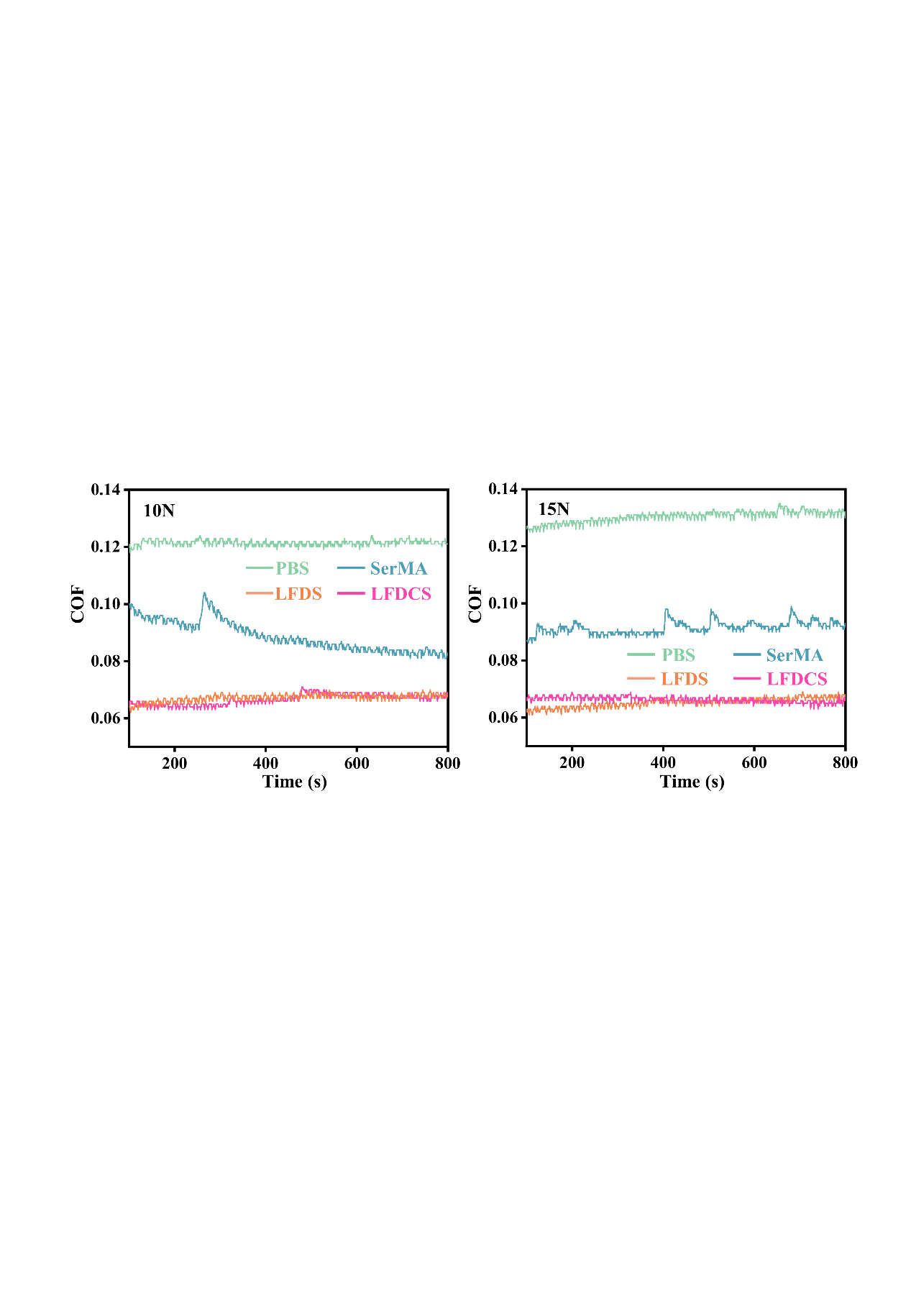


**Supplementary Figure 5.** COF-times curves for 10 and 15N loads.


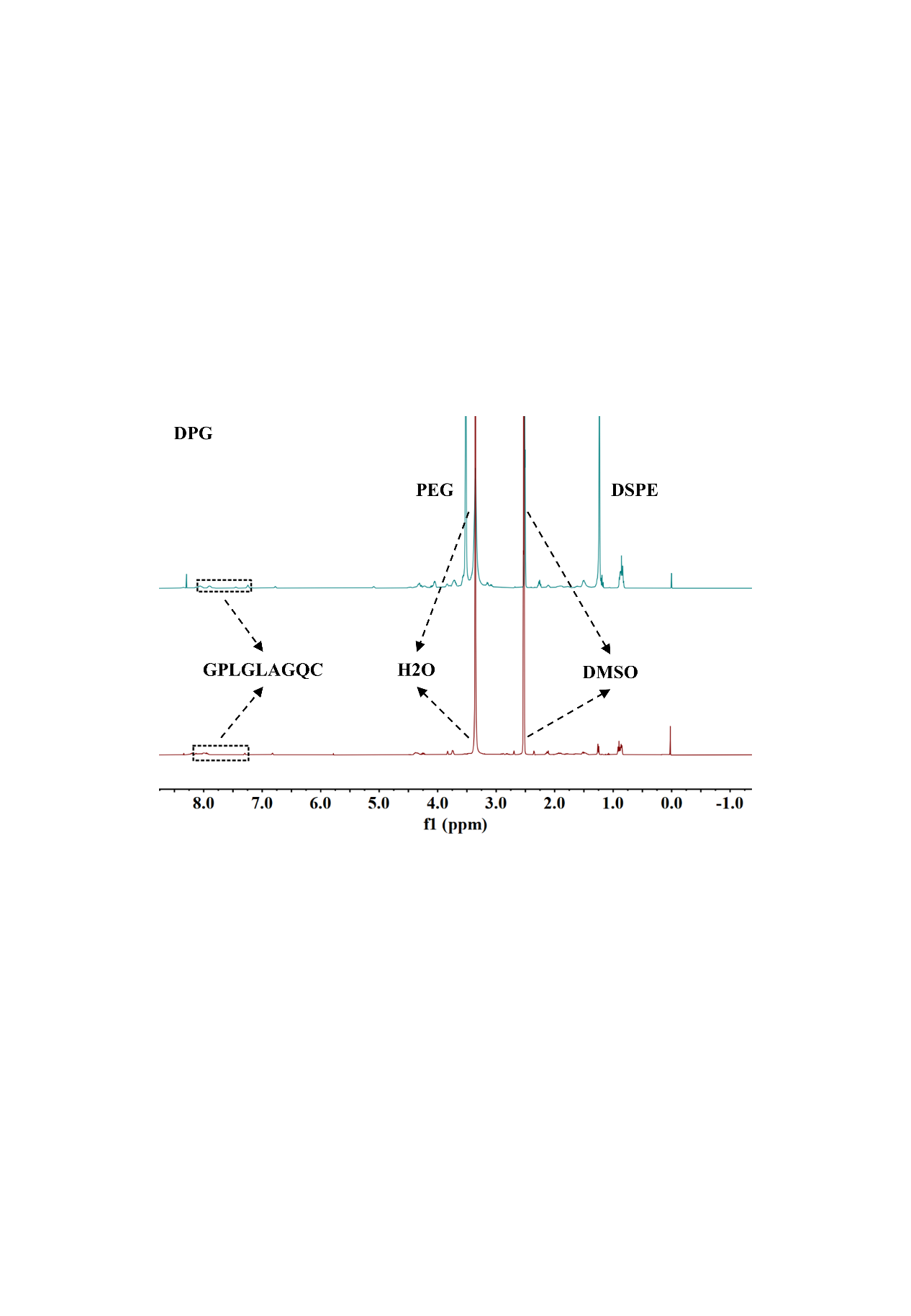


**Supplementary Figure 6.** Nuclear magnetic resonance detection of the structure of DSPE-PEG2k-GPLGLAGQC (DPG).


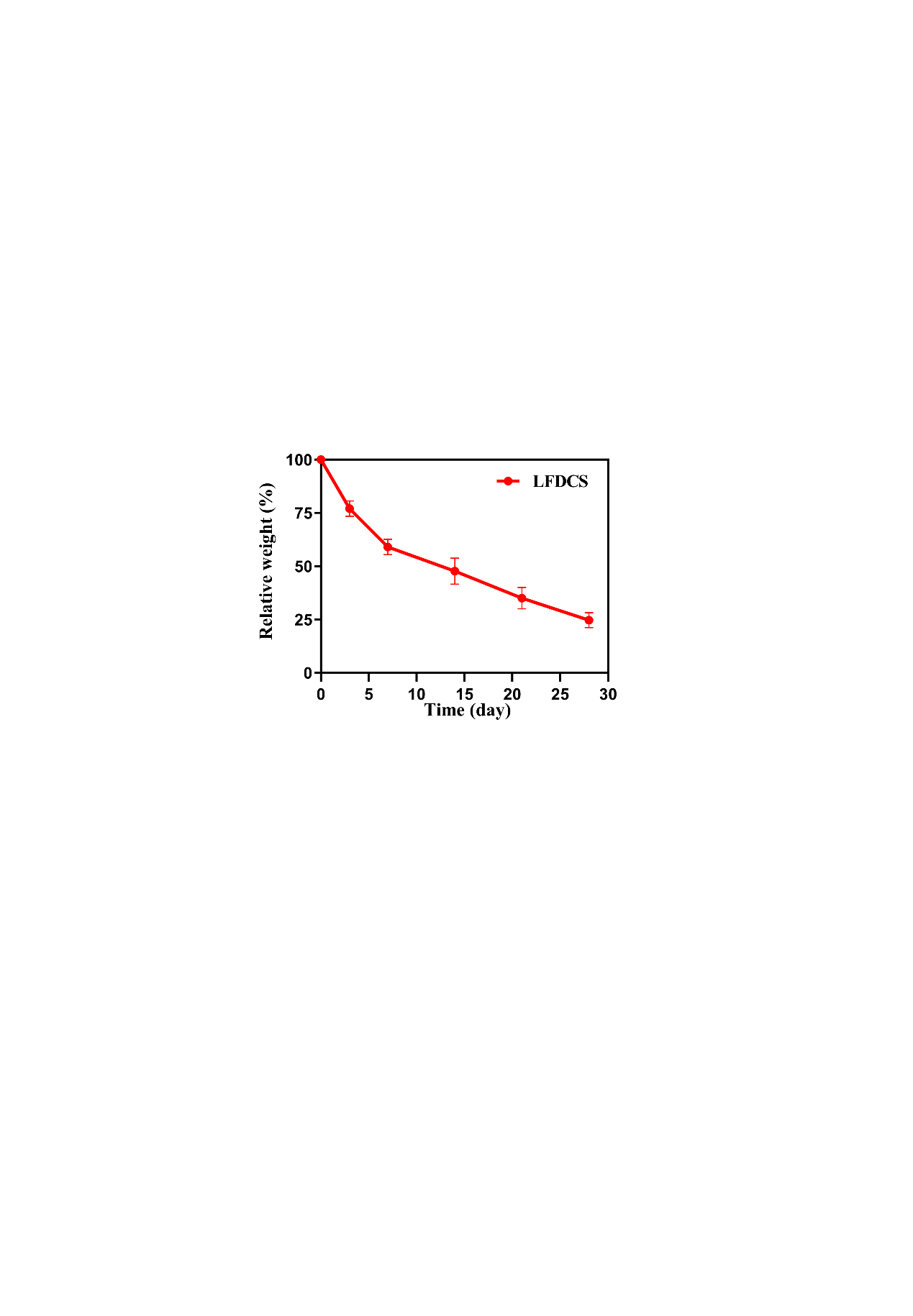


**Supplementary Figure 7.** Degradation of LFDCS (n=3).


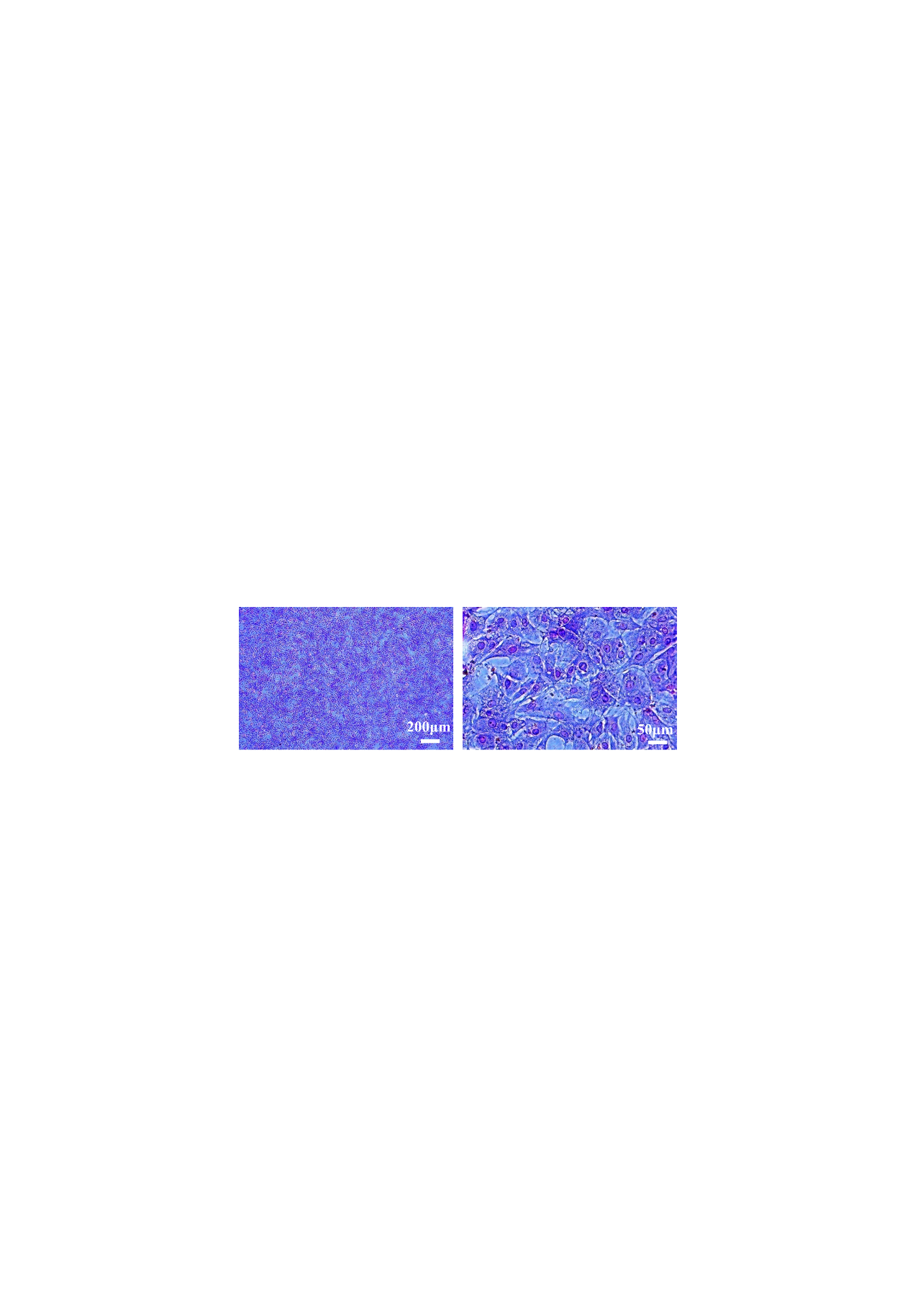


**Supplementary Figure 8.** Toluidine blue staining of chondrocytes.


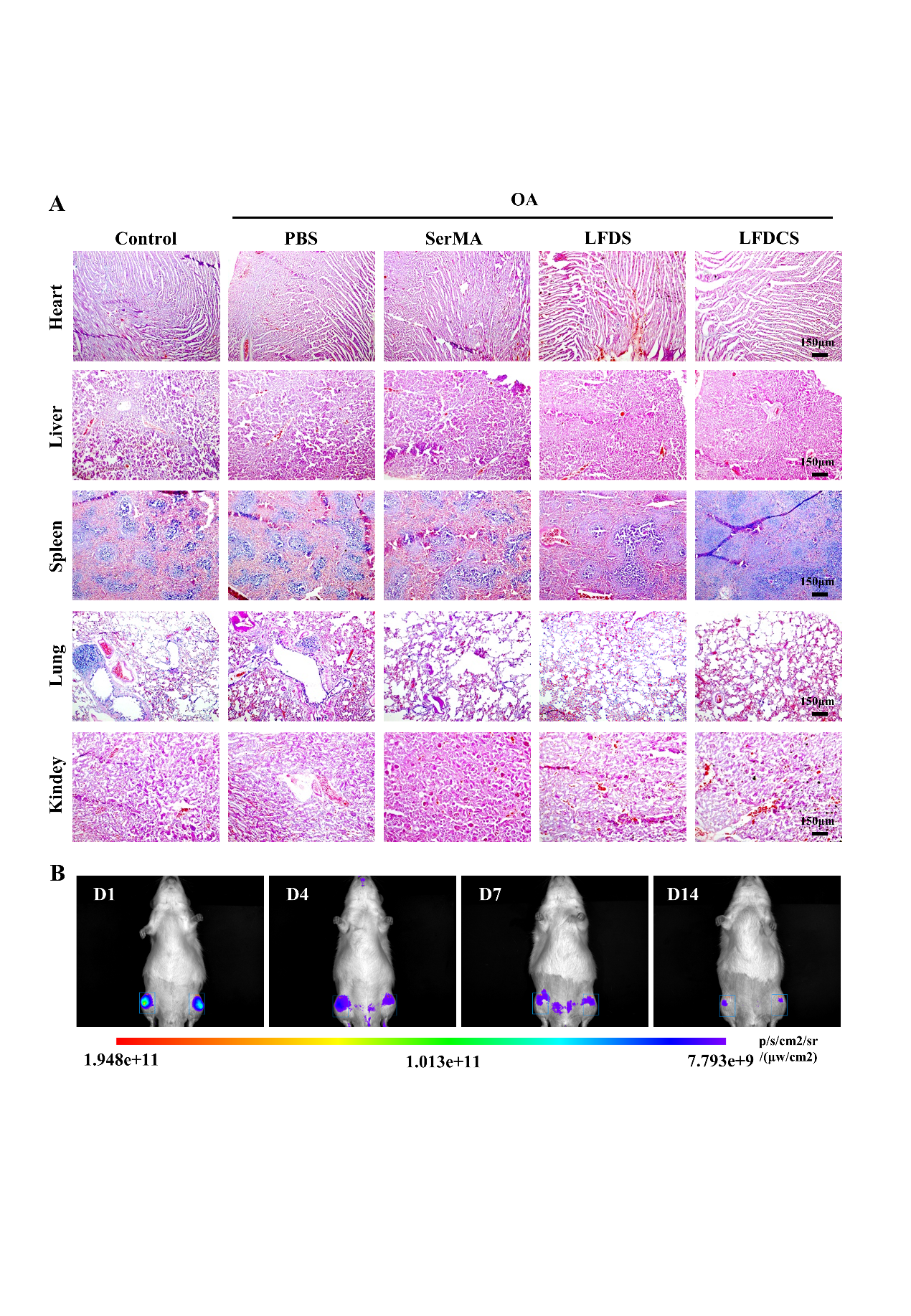


**Supplementary Figure 9.** (A) H&E staining of heart, liver, spleen, lung, and kidney. (B) *In vivo* images of rat knee joints at days 1, 4, 7, and 14 detected by *in vivo* imaging system.


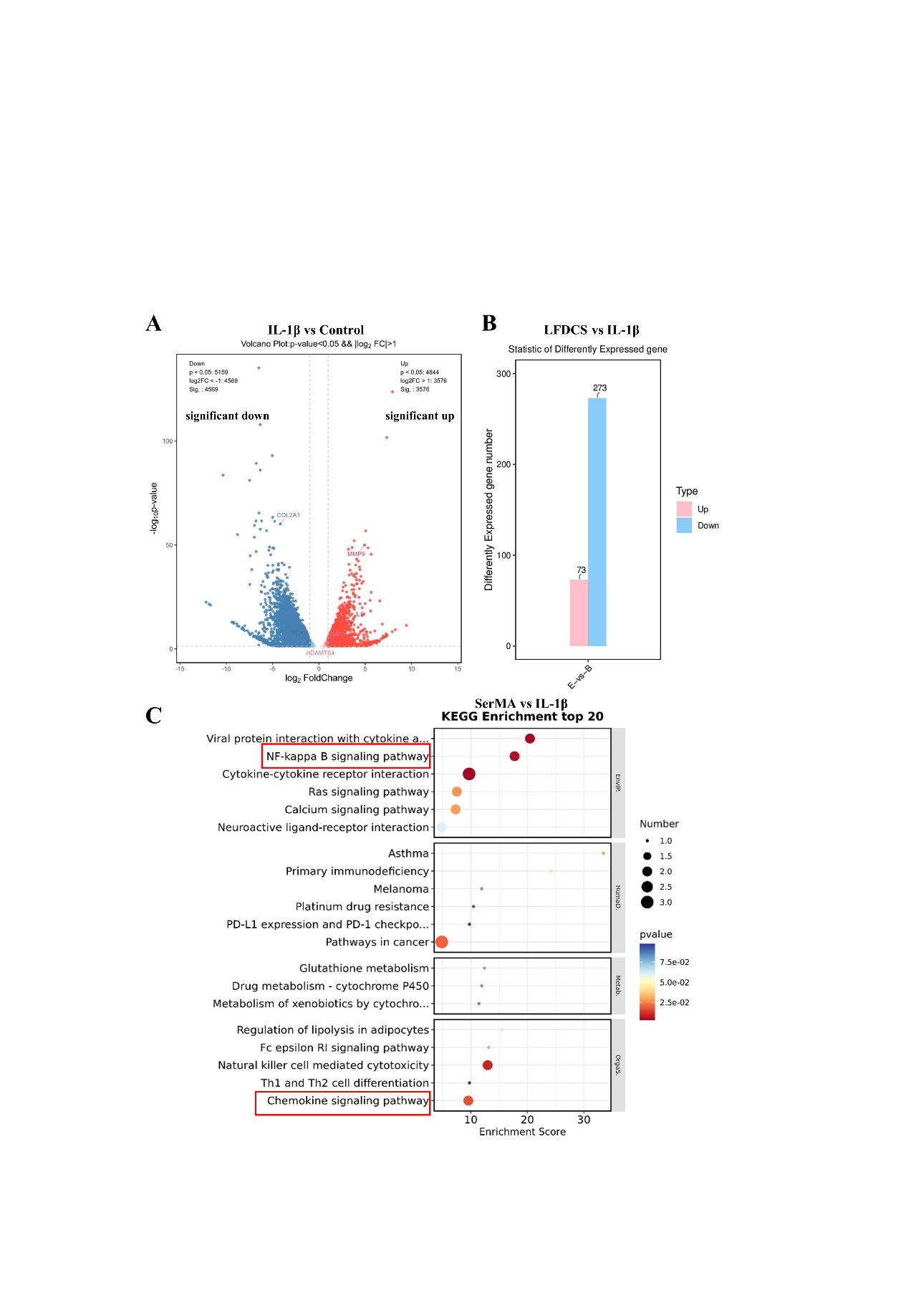


**Supplementary Figure 10.** (A) Volcano map analysis of the differentially expressed genes (DEGs) between IL-1β and Control groups. (B) DEGs between LFDCS and IL-1β groups. (C) KEGG enrichment analysis indicated the down-regulated signaling pathways according to the DEGs. IL-1β: chondrocytes treated with IL-1β; SerMA: chondrocytes treated with IL-1β and the leachate of SerMA hydrogel microspheres; LFDCS: chondrocytes treated with IL-1β and the leachate of LFDCS.

**Supplementary Video 1.** The adhence test of the SerMA microspheres to porcine cartilage.

**Supplementary Table 1. The primers sequence**

| Genes | Forward (5’-3’) | Reverse (5’-3’) |
| --- | --- | --- |
| Mouse-*Inos* | GTTCTCAGCCCAACAATACAAGA | GTGGACGGGTCGATGTCAC |
| Mouse-*Il-1β* | GCAACTGTTCCTGAACTCAACT | ATCTTTTGGGGTCCGTCAACT |
| Mouse-*Tnf-α* | CCCTCACACTCAGATCATCTTCT | GCTACGACGTGGGCTACAG |
| Mouse-*Gapdh* | AGGTCGGTGTGAACGGATTTG | TGTAGACCATGTAGTTGAGGTCA |
| Rat-*Mmp3* | CCTCTGAGTCTTTTCATGGAGGG | ACTTGAGGTTGACTGGTGCC |
| Rat-*Mmp13* | TCCATCCCGAGACCTCATGT | CTCAAAGTGAACCGCAGCAC |
| Rat-*Adamts4* | CTGGTGGTGGCAGATGACAA | GGTTTCGGATGCTTGGATGC |
| Rat-*Sox9* | TTTGCAGTGTTTTCCGCCAC | TGCAGAAGCTTGCGTTGTTC |
| Rat-*Col2* | TGTATGGAAGCCCTCGTCCT | TGCCCCTTTGGCCCTAATTT |
| Rat-*Acan* | TGGGGTCCGTGGGCTCACAA | CATTCGCACGGGAGCAGCCA |
| Rat-*Il-6* | GAAAATCTGCTCTGGTCTTCTGGA | CACTAGGTTTGCCGAGTAGACCT |
| Rat-*Cxcl2* | CCTACCAAGGGTTGACTTCAAGAA | GGCTTCAGGGTTGAGACAAACT |
| Rat-*Cxcl3* | ACATCCAGAGCTTGACGGTG | AGCTCAGCTGGACTTGTCAC |
| Rat-*Cxcl6* | ATATCCCGAACCGGTGCTTC | AATGCCAGCAGGGTGAATGA |
| Rat-*Ccl7* | GCCCTGAAGACAGATGCCTG | TTCCTACCCCTTAGGACCGT |
| Rat-*Ccl19* | CCCCATCCCTGGTAACATCG | CGGATGATGCGTTCTACCCA |
| Rat-*Ccl20* | GAGCAGATCAATTCCTGGAGC | GTCAAAGTTGCTTGCTGCTTCT |
| Rat-*Pik3ap1* | CCACTACCCCAACACCATCG | CCCGTGCATCAGTTCCTCTTTA |
| Rat-*Jak2* | CCACCCAATCATGTCTTCCACA | CAAGGAGAGGAGCTTCAGCC |
| Rat-*I1-7* | CTAAATCGTGCTGCTCGCAA | TTTTTCTTCCTTGCTTGTGCAG |
| Rat-*Chuk* | ACACAGAGTTCTGCTCGCTC | TGTGCTAACGTCTCGCCATC |
| Rat-*Gapdh* | AGGTCGGTGTGAACGGATTTG | TGTAGACCATGTAGTTGAGGTCA |
